# Elimination of transmission of onchocerciasis (river blindness) with long-term ivermectin mass drug administration with or without vector control in sub-Saharan Africa: a systematic review and meta-analysis

**DOI:** 10.1016/S2214-109X(24)00043-3

**Published:** 2024-03-11

**Authors:** Nyamai Mutono, Maria-Gloria Basáñez, Ananthu James, Wilma A Stolk, Anita Makori, Teresia Njoki Kimani, T Déirdre Hollingsworth, Andreia Vasconcelos, Matthew A Dixon, Sake J de Vlas, S M Thumbi

**Affiliations:** a Centre for Epidemiological Modelling and Analysis, University of Nairobi, Nairobi, Kenya; b Paul G Allen School for Global Health, Washington State University, Pullman, WA, USA; c MRC Centre for Global Infectious Disease Analysis, Department of Infectious Disease Epidemiology, School of Public Health, Imperial College London, London, UK; d London Centre for Neglected Tropical Disease Research, Department of Infectious Disease Epidemiology, School of Public Health, Imperial College London, London, UK; e Department of Public Health, Erasmus MC, University Medical Center Rotterdam, Rotterdam, Netherlands; f Ministry of Health Kenya, Kiambu Town, Kenya; g Big Data Institute, University of Oxford, Oxford, UK; h Institute of Immunology and Infection Research, University of Edinburgh, Edinburgh, UK

## Abstract

**Background:**

WHO has proposed elimination of transmission of onchocerciasis (river blindness) by 2030. More than 99% of cases of onchocerciasis are in sub-Saharan Africa. Vector control and mass drug administration of ivermectin have been the main interventions for many years, with varying success. We aimed to identify factors associated with elimination of onchocerciasis transmission in sub-Saharan Africa.

**Methods:**

For this systematic review and meta-analysis we searched for published articles reporting epidemiological or entomological assessments of onchocerciasis transmission status in sub-Saharan Africa, with or without vector control. We searched MEDLINE, PubMed, Web of Science, Embase, Cochrane Central Register of Controlled Trials, African Index Medicus, and Google Scholar databases for all articles published from database inception to Aug 19, 2023, without language restrictions. The search terms used were “onchocerciasis” AND “ivermectin” AND “mass drug administration”. The three inclusion criteria were (1) focus or foci located in Africa, (2) reporting of elimination of transmission or at least 10 years of ivermectin mass drug administration in the focus or foci, and (3) inclusion of at least one of the following assessments: microfilarial prevalence, nodule prevalence, Ov16 antibody seroprevalence, and blackfly infectivity prevalence. Epidemiological modelling studies and reviews were excluded. Four reviewers (NM, AJ, AM, and TNK) extracted data in duplicate from the full-text articles using a data extraction tool developed in Excel with columns recording the data of interest to be extracted, and a column where important comments for each study could be highlighted. We did not request any individual-level data from authors. Foci were classified as achieving elimination of transmission, being close to elimination of transmission, or with ongoing transmission. We used mixed-effects meta-regression models to identify factors associated with transmission status. This study is registered in PROSPERO, CRD42022338986.

**Findings:**

Of 1525 articles screened after the removal of duplicates, 75 provided 282 records from 238 distinct foci in 19 (70%) of the 27 onchocerciasis-endemic countries in sub-Saharan Africa. Elimination of transmission was reported in 24 (9%) records, being close to elimination of transmission in 86 (30%) records, and ongoing transmission in 172 (61%) records. *I*^2^ was 83·3% (95% CI 79·7 to 86·3). Records reporting 10 or more years of continuous mass drug administration with 80% or more therapeutic coverage of the eligible population yielded significantly higher odds of achieving elimination of transmission (log-odds 8·5 [95% CI 3·5 to 13·5]) or elimination and being close to elimination of transmission (42·4 [18·7 to 66·1]) than those with no years achieving 80% coverage or more. Reporting 15–19 years of ivermectin mass drug administration (22·7 [17·2 to 28·2]) and biannual treatment (43·3 [27·2 to 59·3]) were positively associated with elimination and being close to elimination of transmission compared with less than 15 years and no biannual mass drug administration, respectively. Having had vector control without vector elimination (−42·8 [−59·1 to −26·5]) and baseline holoendemicity (−41·97 [−60·6 to −23·2]) were associated with increased risk of ongoing transmission compared with no vector control and hypoendemicity, respectively. Blackfly disappearance due to vector control or environmental change contributed to elimination of transmission.

**Interpretation:**

Mass drug administration duration, frequency, and coverage; baseline endemicity; and vector elimination or disappearance are important determinants of elimination of onchocerciasis transmission in sub-Saharan Africa. Our findings underscore the importance of improving and sustaining high therapeutic coverage and increasing treatment frequency if countries are to achieve elimination of onchocerciasis transmission.

**Funding:**

The Bill & Melinda Gates Foundation and Neglected Tropical Diseases Modelling Consortium, UK Medical Research Council, and Global Health EDCTP3 Joint Undertaking.

**Translations:**

For the Swahili, French, Spanish and Portuguese translations of the abstract see Supplementary Materials section.

**Research in context**

**Evidence before this study**

We searched PubMed and PROSPERO using the terms “onchocerciasis” OR “river blindness” AND “ivermectin” AND “mass drug administration” AND “review” for studies published from database inception to May 18, 2023. Although individual country-specific reviews have been undertaken to assess progress of onchocerciasis elimination programmes, a comprehensive systematic review and meta-analysis to evaluate transmission status after prolonged interventions has not been conducted.

**Added value of this study**

To our knowledge, this is the first systematic review and meta-analysis, and the most comprehensive assessment of factors associated with elimination of *Onchocerca volvulus* transmission in sub-Saharan Africa. Our study shows that a minimum of 10 years of continuous ivermectin mass drug administration with a therapeutic coverage of at least 80% of the eligible population, treatment duration of 15 or more years, and having had biannual (6-monthly) treatment frequency were positively and significantly associated with meeting the criteria for elimination and being close to elimination of transmission. A baseline endemicity level characterised by a microfilarial prevalence of 80% or more (indicative of holoendemicity) and having had vector control (but not vector elimination) were negatively and statistically significantly associated with reporting elimination and being close to elimination of transmission. Some articles that reported elimination of transmission, particularly in east Africa, also described long-lasting declines or disappearance of the blackfly vectors due to environmental change or vector elimination efforts.

**Implications of all the available evidence**

Elimination of transmission or being close to elimination of transmission was reported in 110 (39%) of 282 records retrieved, an important metric regarding progress towards the WHO 2030 target of ending *O volvulus* transmission in endemic countries. Mass drug administration programme duration, therapeutic coverage, treatment frequency, baseline endemicity, and long-lasting reductions in vector density or absence due to vector elimination or secular changes are important contributors to elimination of transmission. Our results could provide data inputs for onchocerciasis transmission modelling studies and could improve understanding of the determinants of the elimination of onchocerciasis transmission.

## Introduction

Despite nearly 50 years of control programmes, onchocerciasis (river blindness) persists in many endemic foci, and is a major risk factor for ocular and skin disease, and epilepsy.^1^, ^2^, ^3^, ^4^ Caused by the filarial nematode *Onchocerca volvulus* and transmitted among humans through blackfly (*Simulium*) vector bites, its estimated global disease burden was 1·23 million disability-adjusted life-years in 2019, with more than 99% of cases in sub-Saharan Africa.^3^

In sub-Saharan Africa, controlling the transmission of onchocerciasis has relied on interventions against blackfly larval stages and mass drug administration of ivermectin to endemic communities. The former aims to decrease vector density and the latter to reduce morbidity, with both impacting transmission. Ivermectin kills microfilariae (the stage transmitted to vectors and mostly responsible for clinical manifestations), and is assumed to reduce adult worm fecundity over time.^5^, ^6^ These strategies have been implemented through two major programmes in Africa: the Onchocerciasis Control Programme in west Africa (OCP, 1974–2002), which covered 11 countries, and the African Programme for Onchocerciasis Control (APOC, 1995–2015), which covered 20 additional countries, and provided technical support to former OCP countries.^7^ At APOC's closure, control and elimination efforts have been continued by national programmes supported by the Expanded Special Project for Elimination of Neglected Tropical Diseases.^8^

The OCP implemented weekly aerial larviciding of blackfly breeding sites in savannah areas of initially seven and finally 11 west African countries. Following the approval of ivermectin for human treatment in 1987, mass treatment of at-risk populations was conducted. Drug distribution evolved from mobile delivery to community-based and ultimately to community-directed treatment with ivermectin.^7^ APOC pioneered community-directed treatment with ivermectin in mesoendemic and hyperendemic areas in sub-Saharan Africa (with a microfilarial prevalence ≥40%, equivalent to a palpable onchocercal nodule prevalence ≥20%).^9^ Ivermectin is predominantly distributed annually, exempting pregnant women, mothers breastfeeding a child younger than 1 week, and children younger than 5 years.^7^ Hypoendemic areas co-endemic with loiasis (a filariasis caused by *Loa loa* and transmitted by tabanid flies) are excluded from ivermectin mass drug administration due to the risk of severe adverse events when individuals with heavy *L loa* microfilaraemia are treated.^7^

Success in onchocerciasis elimination following 15–17 years of annual or 6-monthly ivermectin mass drug administration without vector control was reported in some foci in Mali and Senegal in the Western Extension of the OCP^10^ (a focus is an epidemiological unit recognised as an entity of transmission and thus referred to by endemic countries in their reports and by authors in the published literature). This success led to a paradigm shift from elimination as a public health problem to interruption of transmission through a three-phase approach.^11^, ^12^ The first is the treatment phase, which aims to reach 100% geographical coverage and a therapeutic coverage of 80% or more of the eligible population, with a duration determined by the baseline (pre-intervention) endemicity level, from 13–17 years in areas with moderate endemicity to 20–25 years in highly endemic foci.^12^, ^13^ The second phase comprises conducting surveys to decide whether to stop mass drug administration, which, if indicating that interruption of transmission might have been achieved, are followed by 3–5 years of post-treatment surveillance. The third is the post-elimination surveillance phase, to promptly detect reintroduction and prevent resurgence.^11^, ^12^ In 2016, WHO published guidelines for stopping mass drug administration and verifying interruption of transmission based on serological monitoring of children and molecular xenomonitoring of vectors.^11^

In the past decade, several articles from west, central, and east Africa have reported interruption of onchocerciasis transmission in some foci after at least 15 years of ivermectin mass drug administration.^10^, ^14^, ^15^ In its 2021–30 roadmap on neglected tropical diseases, WHO set a target for verification of interruption of transmission in 12 (31%) of 38 endemic countries by 2030.^16^ With 2030 approaching, a better understanding of factors associated with elimination (interruption) of transmission across sub-Saharan Africa is crucial. We conducted a systematic review and meta-analysis of articles reporting on the status of onchocerciasis transmission after 10 or more years of ivermectin mass drug administration, with or without vector control in sub-Saharan Africa.

## Methods

### Search strategy and selection criteria

We did a systematic review and meta-analysis of all peer-reviewed published articles that had conducted epidemiological or entomological assessments (or both) in onchocerciasis-endemic foci in sub-Saharan Africa after 10 or more years of ivermectin mass drug administration with or without vector control to assess the status of onchocerciasis transmission. We chose articles with 10 or more years of mass drug administration on the basis of an initial literature inspection that indicated no elimination of transmission with shorter treatment programmes. We searched MEDLINE, PubMed, Web of Science, Embase, Cochrane Central Register of Controlled Trials, African Index Medicus, and Google Scholar databases for all articles published from database inception to Aug 19, 2023, without language restrictions. The search terms used were “onchocerciasis” AND “ivermectin” AND “mass drug administration”. Additional search details are in appendix 5 (p 2).

After removing duplicates, three reviewers (NM, AJ, and AM) independently screened abstracts and titles using predefined criteria (appendix 5 p 3). Articles were selected for inclusion if the abstract or title described elimination of transmission or 10 or more years of ivermectin mass drug administration in sub-Saharan African foci. Conflicts between the three reviewers were resolved by two independent reviewers (M-GB and WAS). Full-text articles were retrieved using PubMed, Google, and the libraries of the University of Nairobi (Kenya), Erasmus MC Rotterdam (Netherlands), and Imperial College London (UK). Articles whose full-text manuscript could not be retrieved were excluded. Four independent reviewers (NM, AJ, AM, and TNK) evaluated full-text articles using inclusion and exclusion criteria. The three inclusion criteria were (1) focus or foci located in Africa, (2) reporting of elimination of transmission or at least 10 years of ivermectin mass drug administration in the focus or foci, and (3) inclusion of at least one of the following epidemiological or entomological assessments: microfilarial prevalence, nodule prevalence, Ov16 antibody seroprevalence, and blackfly infectivity prevalence. Epidemiological modelling studies and reviews were excluded. Articles were excluded if they did not meet the two study inclusion criteria, or if they met at least one of the study exclusion criteria. Any discrepancies in the excluded full-text articles were resolved by M-GB and WAS. Study authors were only contacted when we could not find the full paper online and we did not request any individual-level data. We did not include grey literature or any unpublished studies. No ethics approval was required for this study.

### Data extraction and quality assessment

Four reviewers (NM, AJ, AM, and TNK) extracted data in duplicate from the full-text articles. We developed an Excel data collection tool that the reviewers completed as they extracted data. This tool also included a section for reviewers to leave any important comments about each paper to ensure it was easy for one paper to be extracted by more than one author. Information on the study location (country and focus, district, or village), ecological features (savannah, forest, or forest–savannah mosaic), study period, study population, onchocerciasis endemicity, and control and elimination efforts was extracted for each article. Endemicity indicators included baseline microfilarial prevalence, nodule prevalence (calculated using the Rapid Epidemiological Mapping of Onchocerciasis guidelines),^9^ and community microfilarial load (the geometric mean number of microfilariae per skin snip in those aged ≥20 years).^17^ The number of years and frequency of ivermectin mass drug administration, together with the therapeutic coverage of the eligible population were recorded. In addition, information was recorded on simuliid vector species, annual biting rate (number of bites per person per year), annual transmission potential (number of L3 larvae per person per year), blackfly infection (with L1–L3 larvae) or infectivity (with L3 larvae), and vector control type and duration, where available. Vector elimination or declines in vector density due to vector control or environmental change were also recorded when reported. Details on the diagnostic tests used, and the number of positive and total samples tested were recorded (eg, Ov16 seropositivity measured either by IgG4 ELISA or rapid diagnostic test). Other study characteristics such as co-endemicity with other filariases (ie, loiasis or lymphatic filariasis) and civil unrest during the control programme were recorded. Details of the variables extracted are shown in appendix 5 (p 3).

We adopted the STROBE^18^ and the National Institutes of Health guidelines for quality assessment of observational and cross-sectional studies^19^ to assess the quality of articles included in the full-text review (undertaken by NM and AM). A detailed quality assessment for each article is shown in appendix 5 (pp 4–6).

### Data analysis

We used funnel plots to assess risk of bias of the included studies (appendix 5 p 8). We categorised the records from the identified foci as reporting: (1) elimination of transmission, (2) being close to elimination, or (3) experiencing ongoing transmission. Elimination of transmission was considered achieved if the serology threshold (95% upper confidence limit of Ov16 seroprevalence was <0·1% in at least 2000 children aged <10 years using ELISA or rapid diagnostic test) or the entomology threshold (95% upper confidence limit <0·05% vector infectivity in at least 6000 blackflies using O-150 PCR), or both, had been met.^11^ Records reporting elimination of transmission (and post-treatment surveillance) according to the operational parasitological and entomological thresholds proposed by APOC (2010),^20^ were also classified as achieving elimination of transmission.^10^ Records reporting disappearance of blackflies (due to vector control or ecological change) that had achieved the serological threshold were also categorised as having reached elimination of transmission. We adapted APOC's criteria^20^ and used the value of less than 1% microfilarial prevalence to categorise records as being close to elimination of transmission when entomological evaluations or post-treatment surveillance had not been reported. Records reporting microfilarial prevalence of 1% or more were classified as with ongoing transmission.

For articles reporting parasitological data when assessing transmission status post-intervention, we calculated the pooled microfilarial prevalence with 95% CIs using random-effects models, stratified by category of transmission (reported elimination of transmission, close to elimination of transmission, or ongoing transmission) with or without vector control. We used funnel plots to assess systematic heterogeneity and publication bias.^21^ Selected articles differed in terms of location, populations, and other characteristics, potentially introducing heterogeneity. For the random-effects models, τ^2^ was used as a measure of between-study variance. Heterogeneity among articles was measured using the *I*^2^ statistic (the percentage of variation across articles that is due to heterogeneity rather than to chance).^22^ The values of these metrics across articles are shown in appendix 5 p 7; the funnel plots are shown in appendix 5 p 8.

We did a multivariable meta-analysis with non-independent effect sizes (log-odds)^23^ and implemented a mixed-effects logistic regression model (with subnational level or focus as a random effect) to investigate the association between achieving elimination of transmission and being close to elimination of transmission (as a single category) versus ongoing transmission and the following explanatory variables: (1) vector species (*Simulium damnosum sensu lato* complex or *Simulium neavei* group); (2) baseline endemicity (hypoendemic, mesoendemic, hyperendemic, or holoendemic); (3) having a history of vector control (yes or no); (4) number of years of ivermectin treatment (<15, 15–19, or >19); (5) number of years of continuous therapeutic coverage at 80% or more of the eligible population (none, <10, or ≥10); (6) whether foci had ever received biannual treatment (yes or no); and (7) co-endemicity with other filariases (not reported, with loiasis, or with lymphatic filariasis). Baseline endemicity categories were: hypoendemicity (microfilarial prevalence <40% or nodule prevalence <20%); mesoendemicity (microfilarial prevalence ≥40% but <60%, or nodule prevalence ≥20% but <40%); hyperendemicity (microfilarial prevalence ≥60% but <80%, or nodule prevalence ≥40%); and holoendemicity (microfilarial prevalence ≥80%).^9^ We also explored the association between these variables and achieving elimination of transmission versus being close to elimination and ongoing transmission (as a single category). Variables with a p value less than 0·2 at the univariable model step were included in the multivariable model. Stepwise Akaike Information Criterion was used for model selection. The analysis was conducted using R version 4.2.3.^24^

The conduct of the review followed methods previously described.^25^ We also followed the PRISMA guidelines.^26^ This study is registered in PROSPERO, CRD42022338986.

### Role of the funding source

The funders of the study had no role in study design, data collection, data analysis, data interpretation, or writing of the report.

## Results

**Figure 1:**
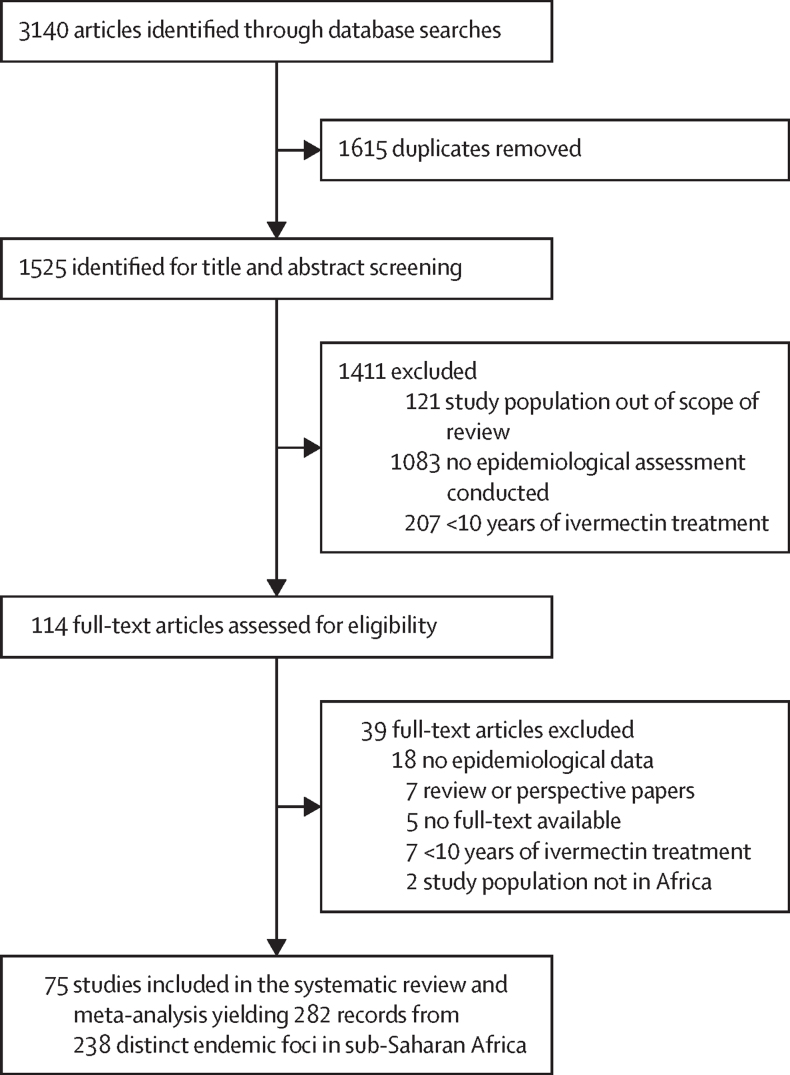
Study selection

**Figure 2:**
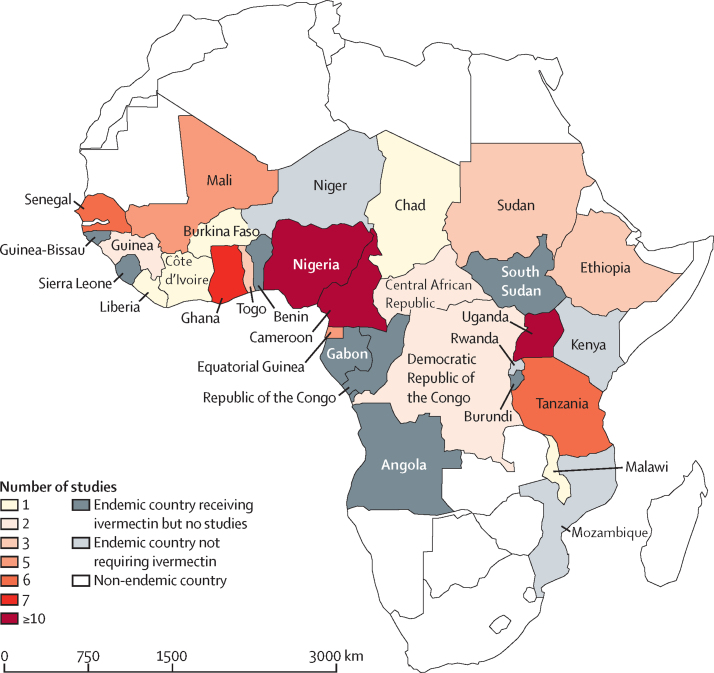
Countries endemic for onchocerciasis, endemic but not requiring ivermectin mass drug administration,^27^ and number of studies from each country

Of the 3140 articles identified, 1525 were screened after removing duplicates (figure 1). Of these, 1411 (93%) were excluded and 114 full-text articles were assessed. A further 39 (3%) articles were excluded during full-text review, with 75 (5%) articles meeting the inclusion criteria and used in the systematic review and meta-analysis. These articles covered 19 (70%) of the 27 endemic countries in sub-Saharan Africa that require ivermectin mass drug administration^27^ (figure 2). 69 (92%) of 75 articles were from single countries, with 44 (64%) of 69 focusing on Cameroon (n=18), Nigeria (n=17), and Uganda (n=9). Six (8%) articles covered more than one country (eg, Senegal, Mali, and Cameroon). The mean interval between data collection (1996–2019) and publication was 3·1 years (SD 1·9). The overall variability between studies was 83·3% (95% CI 79·7–86·3); subgroup variability is shown in appendix 5 (pp 23–24).

The articles provided a total of 282 records from 238 distinct foci, comprising 91 (32%) records from former OCP countries and 191 (68%) from former APOC countries. The table summarises the variables extracted from each record (and the proportion of records per category of the variable) regarding ecological features; vector species; baseline endemicity (and associated variables); vector control; duration, therapeutic coverage, and frequency of ivermectin mass drug administration; co-endemicity with other filariases; and reported onchocerciasis transmission status.

**Table:** Variables extracted from each of the 282 records from 238 foci

		**Total number of records (n=282)**	**Elimination of transmission (n=24)**	**Close to elimination of transmission (n=86)**	**Ongoing transmission (n=172)**
Ecological features		n=169	n=8	n=40	n=121
	Forest	91 (54%)	5 (63%)	22 (55%)	64 (53%)
	Savannah	41 (24%)	3 (38%)	17 (43%)	21 (17%)
	Forest–savannah mosaic	37 (22%)	0	1 (3%)	36 (30%)
Vector species		n=207	n=24	n=71	n=112
	*Simulium damnosum sensu lato* complex	198 (96%)	17 (71%)	71 (100%)	110 (98%)
	*Simulium neavei* group	9 (4%)	7 (29%)	0	2 (2%)
Baseline endemicity		n=202	n=13	n=52	n=137
	Hypoendemic	34 (17%)	3 (23%)	9 (17%)	22 (16%)
	Mesoendemic	54 (27%)	2 (15%)	21 (40%)	31 (23%)
	Hyperendemic	83 (41%)	6 (46%)	20 (38%)	57 (42%)
	Holoendemic	31 (15%)	2 (15%)	2 (4%)	27 (20%)
Baseline microfilarial prevalence		n=127	n=12	n=24	n=91
	Mean (SD; range)	59·9% (22·7; 4–99)	56·6% (21·6; 24–92)	50·9% (20·3; 4–85)	62·7% (23·0; 5–99)
Baseline nodule prevalence		n=116	n=6	n=31	n=79
	Mean (SD; range)	48·6% (22·8; 4–96)	38·5% (19·0; 12–59)	41·3% (17·4; 15–87)	52·3% (24·1; 4–96)
Baseline community microfilarial load per skin snip		n=87	n=7	n=24	n=56
	Mean (SD; range)	21·2 (18·7; 0·1–80·0)	31·3 (16·5; 9·7–49·0)	15·5 (12·9; 0·1–38·6)	22·4 (20·4; 0·2–80·0)
Baseline annual biting rate, bites per person per year		n=26	Not available	n=4	n=22
	Range	868 to >80 000	Not available	868 to 26 400	868 to >80 000
Baseline annual transmission potential, L3 per person per year		n=25	Not available	n=2	n=23
	Mean (SD; range)	1504 (3364; 11–16 900)	Not available	190 (109; 113–267)	1617 (3490; 113–16 900)
Vector control reported		n=282	n=24	n=86	n=172
	Yes (in foci under OCP)	59 (21%)	0	17 (20%)	40 (23%)
	Yes (in foci under APOC)	15 (5%)	6 (25%)	7 (8%)	2 (1%)
	No	208 (74%)	18 (75%)	62 (72%)	130 (76%)
Number of years of vector control		n=65	n=6	n=17	n=42
	Mean (SD; range)	9·1 (7·5; 1–31)	3·7 (2·4; 1–7)	8·4 (7·5; 1–28)	10·1 (7·8; 2–31)
Duration of ivermectin mass drug administration, years		n=260	n=22	n=76	n=164
	Mean (SD; range)	16·3 (5·1; 10–30)	19·0 (4·6; 10–28)	17·2 (5·1; 10–29)	15·5 (4·9; 10–30)
Therapeutic coverage (of eligible individuals) during the intervention period		n=195	n=20	n=62	n=114
	<80%	98 (50%)	0	43 (69%)	55 (48%)
	≥80%	97 (50%)	20 (100%)	19 (31%)	59 (52%)
Number of years with reported therapeutic coverage maintained at ≥80%		n=71	n=19	n=16	n=36
	Mean (SD; range)	7·7 (6·2; 1–22)	14·7 (5·6; 2–22)	5·4 (4·5; 1–16)	5·1 (3·9; 1–13)
Proportion of eligible population* never treated in the past 5–10 rounds		n=30	n=0	n=2	n=28
	Mean (SD; range)	20% (10·1; 1–41)	Not available	31% (27·6; 11–50†)	20% (9·5; 1–41)
Biannual ivermectin mass drug administration		n=282	n=24	n=86	n=172
	Never had (only annual mass drug administration)	261 (93%)	18 (75%)	79 (92%)	164 (95%)
	Have had biannual mass drug administration	21 (7%)	6 (25%)	7 (8%)	8 (5%)
Co-endemicity with other filariases		n=282	n=24	n=86	n=172
	Not reported	236 (84%)	15 (63%)	64 (74%)	157 (91%)
	With loiasis	11 (4%)	0	8 (9%)	3 (2%)
	With lymphatic filariasis	35 (12%)	9 (38%)	14 (16%)	12 (7%)

APOC=African Programme for Onchocerciasis Control. OCP=Onchocerciasis Control Programme.

* Not all studies assessed population aged ≥5 years.

† 50% systematic non-adherence recorded in Bioko, but close to elimination of transmission (<1% microfilarial prevalence) likely because vector was eliminated in 2005.^15^

Of the 282 records, 24 (9%) reported elimination of transmission, 86 (30%) were classified as close to elimination of transmission, and 172 (61%) had ongoing transmission. Eight of the 24 records reporting elimination of transmission were from Uganda (from the foci of Imaramagambo, Itwara, Kashoya-Kitomi, Mpamba-Nkusi, Mount Elgon, Wadelai, and Wambabya-Rwamarongo, with *S neavei*,^28^, ^29^, ^30^, ^31^, ^32^, ^33^, ^34^ and Obongi with *S damnosum sensu lato*),^14^ seven were from Nigeria (Kaduna, Kebbi, Zamfara, Plateau, and Nasarawa States),^35^, ^36^, ^37^ two were from Sudan (Abu Hamed and Galabat),^38^, ^39^, ^40^ two were from Mali (River Bakoye), one straddling Senegal and Mali (River Falémé), two from Senegal (River Gambia),^10^, ^41^ one from Equatorial Guinea (Bioko Island),^15^ and one from Ethiopia (Metema),^40^ with *S damnosum sensu lato* (in Metema, an entomological hot-spot was detected along the Wudi Gemzu river and mass drug administration implemented quarterly^40^). Ten (42%) of the 24 records reporting elimination of transmission reported having received biannual mass drug administration.^10^, ^30^, ^31^, ^32^, ^33^, ^34^, ^38^, ^39^, ^40^ For River Bakoye, River Falémé, and River Gambia, onchocerciasis elimination was reported^41^ (before the WHO guidelines)^11^ using microfilarial prevalence of less than 1% and blackfly infectivity rate of less than 0·05%.^10^, ^20^ Three of the 24 records reporting elimination of transmission (Kashoya-Kitomi, Obongi, and Bioko Island) stated that ivermectin mass drug administration had continued for 4–14 years after the vector had been eliminated or disappeared due to vector control or ecological and environmental change.^14^, ^15^, ^30^ Post-treatment surveillance was reported in eight (33%) records reporting elimination of transmission,^10^, ^28^, ^34^, ^37^, ^39^ six (25%) of which were in Uganda^42^ (appendix 5 pp 10–22). A histogram of the number of times records entered the analysis according to their transmission status at the time of the study is shown in appendix 5 (p 9).

**Figure 3:**
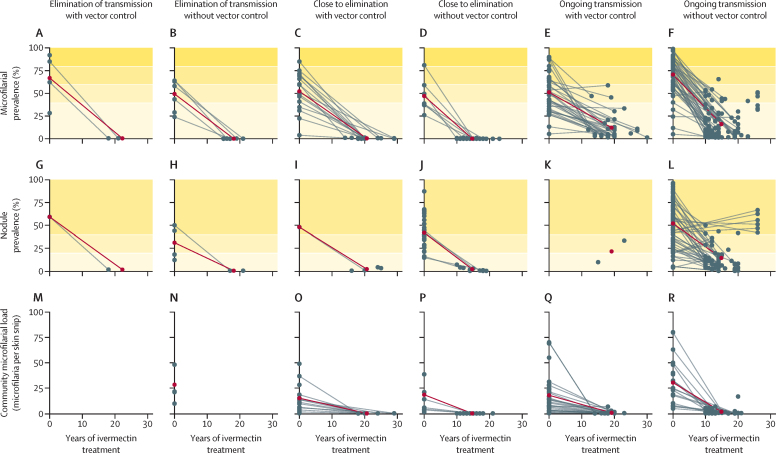
Infection trends in records reporting elimination of transmission, close to elimination of transmission, and ongoing transmission (A–F) The baseline (pre-control) and final values of microfilarial prevalence. (G–L) The baseline (pre-control) and final values of nodule prevalence. (M–R) The baseline (pre-control) and final values for community microfilarial load. All data are for records that reported 10 or more years of ivermectin treatment with or without vector control. The shaded horizontal bands in A–F show baseline endemicity levels according to microfilarial prevalence, from hypoendemic (very light yellow) to holoendemic (dark yellow). The shaded bands in G–L indicate endemicity levels according to nodule prevalence, from hypoendemic (very light yellow) to hyperendemic (medium yellow). The grey lines indicate the individual records. The dark red lines represent the mean values at baseline on the vertical axis and at the end of the intervention period reported on the horizontal axis (in some articles, only baseline infection data were provided, while others reported duration of ivermectin mass drug administration was not accompanied by parasitological data at the end of the intervention period).

Endemicity for 126 records, including parasitological information (microfilarial or nodule prevalence and community microfilarial load) at baseline and at the time epidemiological evaluations took place are shown in figure 3. Pre-control microfilarial prevalence ranged from 3·9% (95% CI 2·0–6·7) to 98·9% (97·1–99·6). The mean pre-intervention microfilarial prevalence for those records reporting elimination of transmission with vector control (in a mean of 22 years [SD 4]; figure 3A) was 66·9% (SD 28·7, range 28·3–92·0), whereas for those reporting elimination of transmission without vector control (in a mean of 17 years [SD 5]; figure 3B), this value was 49·3% (SD 17·2, range 24·0–64·0). Records classified as close to elimination of transmission with vector control in a mean of 21 years (SD 4) had mean pre-intervention microfilarial prevalence of 52·2% (SD 21·7, range 3·9–85·0); those close to elimination of transmission without vector control in a mean of 15 years (SD 3) had microfilarial prevalence of 47·0% (SD 18·2, range 26·0–81·0; figure 3C, D). A similar pattern was observed for nodule prevalence (figure 3G–J). For records with ongoing transmission, the mean baseline microfilarial prevalence was 51·2% (SD 21·2; range 5·3–90·0) with vector control after a mean of 19 years (SD 4, range 10–30) of treatment reported, and 70·7% (SD 20·4, range 5·0–98·9) without vector control after a mean of 15 years (SD 5, range 10–27) of treatment reported (figure 3E, F). The range for baseline community microfilarial load was 0·2–70·0 microfilariae per skin snip in records with ongoing transmission with vector control (figure 3Q), and 4·8–80·0 microfilariae per skin snip in those without vector control (figure 3R), and was generally greater than in records with elimination of transmission and close to elimination of transmission than those with ongoing transmission (figure 3M–P). During the intervention period, the records reporting ongoing transmission had a mean nodule prevalence of 21·5% (SD 16·8, range 9·6–33.3) with vector control and 14·2% (SD 16·4, range 0·0–66·7) without vector control (figure 3K, L).

At the time epidemiological evaluations were conducted to assess intervention impact, the microfilarial prevalence among records reporting elimination of transmission was 0·3% (95% CI 0·2–0·6) with vector control and 0·1% (0·1–0·3) without vector control, indicating that these values were not significantly different. Records reporting being close to elimination of transmission with vector control had microfilarial prevalence of 0·4% (0·2–0·9), significantly higher than 0·0% (0·0–0·1) for those without vector control. In records reporting ongoing transmission and vector control, microfilarial prevalence was 8·1% (5·6–11·5) and in those without vector control was 9·7% (7·6–12·2), which was not significantly different. The study variance and heterogeneity for each of these results, detailing the microfilarial prevalence reported for each record, are shown in appendix 5 (pp 23–24).

**Figure 4:**
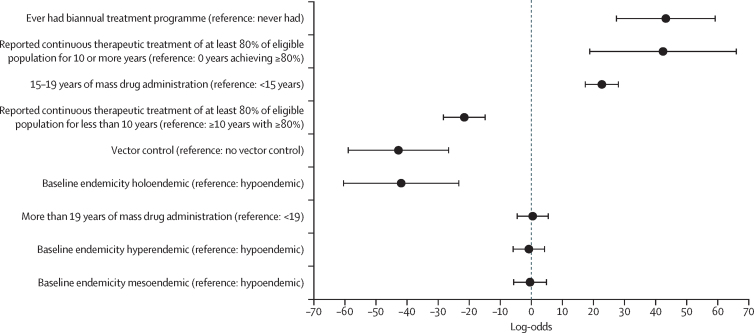
Results of the final multivariable logistic model Figure shows the log-odds of factors associated with elimination of transmission or being close to elimination combined among identified records from onchocerciasis foci in sub-Saharan Africa. Black circles indicate the log-odds values; the horizontal error bars are 95% CIs.

The final multivariable meta-regression models showed that the following variables were positively and significantly associated with higher log-odds of achieving elimination of transmission and being close to elimination of transmission analysed together: having had biannual treatment (log-odds 43·3 [95% CI 27·2 to 59·3]) compared with never having had biannual treatment (only annual); having 10 or more years of continuous treatment with a reported coverage of 80% or more of the eligible population (42·4 [18·7 to 66·1]) compared with no years achieving a coverage of 80% or more; and reporting 15–19 years of ivermectin mass drug administration (22·7 [17·2 to 28·2]) compared with less than 15 years. By contrast, reporting less than 10 years of treatment at 80% or more coverage of the eligible population (log-odds −21·6 [95% CI −28·4 to −14·7]) compared with 10 years or more at such coverage, having had vector control without vector elimination (−42·8 [−59·1 to −26·5]) compared with no vector control, and baseline holoendemicity (−41·9 [−60·6 to −23·2]) compared with hypoendemicity, were significantly associated with an increased risk of experiencing ongoing transmission (figure 4). The only variable that was positively and significantly associated with reporting elimination of transmission compared with being close to elimination of transmission and experiencing ongoing transmission analysed together was having 10 or more years of continuous treatment with coverage of 80% or more of the eligible population (log-odds 8·5 [3·5 to 13·5]). Additional multivariable analyses are shown in appendix 5 (p 25).

## Discussion

We reviewed published articles from sub-Saharan Africa where 10 or more years of ivermectin mass drug administration had been implemented, with or without vector control, to identify factors associated with reporting elimination of transmission, being closer to achieving elimination of transmission, or experiencing ongoing onchocerciasis transmission. Of the 282 records, 110 (39%) reported results consistent with elimination of transmission or close to elimination of transmission, an important metric regarding progress towards *O volvulus* elimination as we approach the WHO 2030 target date.^16^ There were no articles from Angola, Benin, Burundi, Republic of the Congo, Gabon, Guinea-Bissau, Sierra Leone, or South Sudan reporting elimination of transmission despite some of these countries having a long history of control (eg, Benin, Sierra Leone, and Guinea-Bissau). Treatment coverage has been low in Angola, South Sudan, and the Democratic Republic of the Congo.^12^ Elimination of transmission as defined by WHO guidelines^11^ was reported in 21 records.^14^, ^15^, ^28^, ^29^, ^30^, ^31^, ^32^, ^33^, ^34^, ^35^, ^36^, ^37^, ^38^, ^39^, ^40^ For three records^10^, ^41^ previous criteria for elimination had been used.^20^

Having had vector control (but not reporting vector elimination) as well as baseline holoendemicity was negatively associated with elimination of transmission and being close to elimination of transmission. Records reporting elimination of transmission with vector control had a higher baseline prevalence than those where vector control had not been implemented. Of the 74 records documenting antivectorial interventions, 59 (80%) were from former OCP countries. In these countries, long-term larviciding of *S damnosum sensu lato* breeding sites was deployed in savannah areas to eliminate onchocercal blindness, mostly associated with high endemicity,^43^ which, from our results, is a determinant factor of ongoing transmission.

Seven (29%) of the 24 records that reported elimination of transmission reported elimination of *S neavei*,^28^, ^29^, ^30^, ^31^, ^32^, ^33^, ^34^ or the Bioko form of *Simulium yahense* (within the *S damnosum* complex)^15^ with vector control, and the remainder without vector control (with *S damnosum sensu lato*,^10^, ^36^, ^37^, ^38^, ^39^, ^40^ or where either or both vectors were present).^14^, ^28^, ^32^, ^33^ In Obongi and Wadelai (Uganda), no simuliids were found at the time epidemiological and entomological evaluations were conducted, and their disappearance (due to ecological or environmental change) is likely to have contributed to elimination of transmission and minimal resurgence.^14^, ^33^ In Abu-Hamed (Sudan), the Merowe Dam had a positive impact on elimination of transmission, as it detrimentally affected the vector population.^39^ In Kebbi and Zamfara (Nigeria) blackfly density was very low at the time of entomological evaluations and was unlikely to sustain transmission (these states, as well as Kaduna, had ranged from hypoendemic to hyperendemic at baseline).^35^, ^44^ In the Amani focus (Tanzania), population declines of *Simulium woodi* (in the *S neavei* group), related to deforestation, decreased onchocerciasis transmission.^45^, ^46^ Some records reported mass drug administration continuing for 4–14 years after vector elimination or disappearance.^14^, ^15^, ^30^, ^35^ Therefore, although vector elimination or declines are likely to have contributed to elimination of transmission, the duration of treatment programmes was prolonged. This particularly applies to areas co-endemic with lymphatic filariasis, which integrated ivermectin mass drug administration for lymphatic filariasis and onchocerciasis,^14^, ^35^, ^36^ making it difficult to ascertain precisely when elimination of transmission was achieved. Where elimination of transmission has been reported, ivermectin had been distributed for 15–26 years.

The odds of achieving elimination of transmission and being close to elimination of transmission were significantly higher for records reporting 10 or more years of continuous treatment with therapeutic coverage of 80% or more of the eligible population. Receiving 15 or more years of ivermectin mass drug administration, as well as having had biannual treatment were positively and significantly associated with elimination of transmission and being close to elimination of transmission. In the Onchocerciasis Elimination Program for the Americas, increasing to 6-monthly (and in some foci to 3-monthly) treatment with coverage of 85% or more of the eligible population led to elimination of transmission in 11 of 13 foci from 1989 to 2016.^47^

Possible explanations for the absence of continuous treatment of at least 80% of the eligible population in some foci include premature halting of interventions, loiasis co-endemicity, civil unrest, and uncoordinated cross-border treatment efforts.^8^, ^48^, ^49^ Stopping interventions prematurely might have resulted in transmission resurgence in Burkina Faso, where treatment was discontinued after 6 years of 4-monthly treatment (Bougouriba focus) without epidemiological assessment, or where larviciding ceased without ivermectin treatment for 2 years (Comoé Valley).^50^, ^51^ Although records that report stopping mass drug administration following WHO's guidelines^11^ did not detect resurgence, implementation of post-treatment surveillance for longer than 3–5 years might be necessary (predominantly where baseline endemicity was high and ecological conditions propitious for high vector density have not changed), as low infection prevalence can be maintained and remain undetected before resurgence becomes evident.^52^ This reported resurgence is supported by a parasitological and serological evaluation in River Gambia (Senegal, co-endemic with lymphatic filariasis), which suggested the possibility of ongoing transmission 5 years after onchocerciasis elimination was deemed to have been achieved.^53^

Whereas harmonised cross-border treatment enhances elimination of transmission,^40^ uncoordinated efforts hamper it.^7^, ^48^ Elimination interventions implemented by one country might be hindered if not matched by a neighbouring country. In Uganda, civil unrest experienced by neighbouring South Sudan and the Democratic Republic of the Congo had a detrimental effect on Uganda's progress towards country-wide elimination.^42^ Civil unrest has contributed to interrupted or slow start of mass drug administration programmes, lower levels of therapeutic coverage, and greater proportion of systematic non-adherence. Although this is especially relevant in central Africa, where forest onchocerciasis predominates, frequently co-endemic with loiasis,^7^, ^48^, ^49^ ivermectin mass drug administration has also been interrupted due to conflict in west Africa (eg, Sierra Leone, where good coverage was only achieved after an 11-year civil conflict, and Côte d'Ivoire, resulting in transmission resurgence).^54^, ^55^

Our study has some limitations. We included peer-reviewed but not grey literature sources. This might partly explain the absence of articles from eight endemic countries. As 68% of records originated from former APOC countries, where hypoendemic areas were not prioritised for ivermectin mass drug administration, the reported number of years required for elimination of transmission mainly pertains to mesoendemic and hyperendemic foci. Another limitation is the absence of explicit assessment of intervention impact in records reporting co-endemicity with lymphatic filariasis because ivermectin mass drug administration was concomitantly distributed or extended in duration (for either disease), making it difficult to assess when precisely during such programmes elimination of onchocerciasis transmission might have been achieved. In River Bakoye and River Falémé, Mali, onchocerciasis elimination was deemed to have been achieved after 15–16 years of annual treatment,^10^, ^41^ albeit not following the updated WHO guidelines,^11^ which were unpublished at the time. When an integrated lymphatic filariasis and onchocerciasis serological evaluation was conducted a decade later, it became apparent that these foci had received 24–25 rounds of ivermectin mass drug administration.^56^ Elimination of onchocerciasis was confirmed (although the upper 95% confidence limit of Ov16 seroprevalence in children aged <10 years was 0·6% in River Bakoye and 0·25% in River Falémé, above the 0·1% threshold proposed).^11^, ^56^

Our findings underscore the importance of improving and sustaining therapeutic coverage if countries are to achieve elimination of onchocerciasis transmission. Despite relying on reported coverage, accurately accessing true coverage remains notoriously difficult due to the dependence on precise population denominators, updated censuses, and ideally, direct observation of treatment. When coverage surveys are conducted, realised coverage is typically lower, and studies robustly quantifying patterns of treatment adherence to ivermectin over multiple treatment rounds are scarce.^57^, ^58^ Seemingly high levels of coverage might mask whether individuals are treated randomly or systematically over multiple rounds. The latter, by reaching the same or similar individuals, leads to never-treated or seldom-treated population subgroups, hindering elimination.^59^ Therefore, we recommend that detailed studies be conducted to evaluate treatment coverage and adherence in communities, collecting information on how many previous rounds individuals have attended, enabling quantification of systematic non-adherence levels.^58^, ^59^, ^60^, ^61^, ^62^ We also recommend that as treatment expands to hypoendemic areas, data on progress to and attainment of elimination of transmission be collected and reported for these foci so our work can be updated with this important information. Since in some foci, efforts to eliminate the vector contributed to achieving or protecting elimination of transmission, we recommend that, where feasible, and to complement mass drug administration, sustainable and preferably community-directed antivectorial measures be implemented with appropriate frequency and duration to interrupt transmission.^63^, ^64^ Our results can be used to provide input data for onchocerciasis transmission modelling studies and to improve understanding of the determinants of elimination of onchocerciasis transmission (eg, modelling secular trends in vector density not captured in current modelling studies, improving the modelling of coverage and adherence, understanding the impact of increased treatment frequency, and improving the modelling of vector control efficacy, frequency, duration, and type in combination with mass drug administration).

## Data sharing

All datasets generated and analysed for this work are available in the main text and appendix 5. The code for data analysis is available at: https://github.com/cema-uonbi/onchocerciasis_systematic_review.git.

## Declaration of interests

We declare no competing interests.

## Supplementary Materials

Swahili translation of the abstract

French translation of the abstract

Spanish translation of the abstract

Portuguese translation of the abstract

## References

[1] Komlan K, Vossberg PS, Gantin RG (2018). *Onchocerca volvulus* infection and serological prevalence, ocular onchocerciasis and parasite transmission in northern and central Togo after decades of *Simulium damnosum* s.l. vector control and mass drug administration of ivermectin. PLoS Negl Trop Dis.

[2] Forrer A, Wanji S, Obie ED (2021). Why onchocerciasis transmission persists after 15 annual ivermectin mass drug administrations in south-west Cameroon. BMJ Glob Health.

[3] Vos T, Lim SS, Abbafati C (2020). Global burden of 369 diseases and injuries in 204 countries and territories, 1990–2019: a systematic analysis for the Global Burden of Disease Study 2019. Lancet.

[4] Chesnais CB, Nana-Djeunga HC, Njamnshi AK (2018). The temporal relationship between onchocerciasis and epilepsy: a population-based cohort study. Lancet Infect Dis.

[5] Basáñez MG, Pion SDS, Boakes E, Filipe JAN, Churcher TS, Boussinesq M (2008). Effect of single-dose ivermectin on *Onchocerca volvulus*: a systematic review and meta-analysis. Lancet Infect Dis.

[6] Plaisier AP, Alley ES, Boatin BA (1995). Irreversible effects of ivermectin on adult parasites in onchocerciasis patients in the Onchocerciasis Control Programme in west Africa. J Infect Dis.

[7] Boatin BA, Richards FO (2006). Control of onchocerciasis. Adv Parasitol.

[8] Colebunders R, Basáñez MG, Siling K (2018). From river blindness control to elimination: bridge over troubled water. Infect Dis Poverty.

[9] Noma M, Zouré HG, Tekle AH, Enyong PA, Nwoke BE, Remme JHF (2014). The geographic distribution of onchocerciasis in the 20 participating countries of the African Programme for Onchocerciasis Control: (1) priority areas for ivermectin treatment. Parasit Vectors.

[10] Traore MO, Sarr MD, Badji A (2012). Proof-of-principle of onchocerciasis elimination with ivermectin treatment in endemic foci in Africa: final results of a study in Mali and Senegal. PLoS Negl Trop Dis.

[11] WHO (2016). Guidelines for stopping mass drug administration and verifying elimination of human onchocerciasis: criteria and procedures. https://www.who.int/publications/i/item/9789241510011.

[12] Tekle AH, Zouré HGM, Noma M (2016). Progress towards onchocerciasis elimination in the participating countries of the African Programme for Onchocerciasis Control: epidemiological evaluation results. Infect Dis Poverty.

[13] Winnen M, Plaisier AP, Alley ES (2002). Can ivermectin mass treatments eliminate onchocerciasis in Africa?. Bull World Health Organ.

[14] Luroni LT, Gabriel M, Tukahebwa E (2017). The interruption of *Onchocerca volvulus* and *Wuchereria bancrofti* transmission by integrated chemotherapy in the Obongi focus, north western Uganda. PLoS One.

[15] Herrador Z, Garcia B, Ncogo P (2018). Interruption of onchocerciasis transmission in Bioko Island: accelerating the movement from control to elimination in Equatorial Guinea. PLoS Negl Trop Dis.

[16] WHO (2021). Ending the neglect to attain the Sustainable Development Goals: a road map for neglected tropical diseases 2021–2030. https://www.who.int/publications/i/item/9789240010352.

[17] Remme J, Ba O, Dadzie KY, Karam M (1986). A force-of-infection model for onchocerciasis and its applications in the epidemiological evaluation of the Onchocerciasis Control Programme in the Volta River basin area. Bull World Health Organ.

[18] von Elm E, Altman DG, Egger M, Pocock SJ, Gøtzsche PC, Vandenbroucke JP (2007). The Strengthening the Reporting of Observational Studies in Epidemiology (STROBE) statement: guidelines for reporting observational studies. PLoS Med.

[19] National Heart, Lung and Blood Institute (2013). Study quality assessment tools. https://www.nhlbi.nih.gov/health-topics/study-quality-assessment-tools.

[20] WHO, African Programme for Onchocerciasis Control (2010). Conceptual and operational framework of onchocerciasis elimination with ivermectin treatment. https://apps.who.int/iris/handle/10665/275466.

[21] Duval S, Tweedie R (2000). Trim and fill: a simple funnel-plot-based method of testing and adjusting for publication bias in meta-analysis. Biometrics.

[22] Higgins JPT, Thompson SG, Deeks JJ, Altman DG (2003). Measuring inconsistency in meta-analyses. BMJ.

[23] Cheung MW-L (2019). A guide to conducting a meta-analysis with non-independent effect sizes. Neuropsychol Rev.

[24] R Core Team (2020).

[25] Tawfik GM, Dila KAS, Mohamed MYF (2019). A step by step guide for conducting a systematic review and meta-analysis with simulation data. Trop Med Health.

[26] Page MJ, McKenzie JE, Bossuyt PM (2021). The PRISMA 2020 statement: an updated guideline for reporting systematic reviews. BMJ.

[27] WHO (2022). Status of endemicity of onchocerciasis. https://www.who.int/data/gho/data/indicators/indicator-details/GHO/status-of-endemicity-of-onchocerciasis.

[28] Katabarwa MN, Katamanywa J, Lakwo T (2016). The Imaramagambo onchocerciasis focus in southwestern Uganda: interruption of transmission after disappearance of the vector *Simulium neavei* and its associated freshwater crabs. Am J Trop Med Hyg.

[29] Lakwo TL, Garms R, Rubaale T (2013). The disappearance of onchocerciasis from the Itwara focus, western Uganda after elimination of the vector *Simulium neavei* and 19 years of annual ivermectin treatments. Acta Trop.

[30] Lakwo T, Garms R, Wamani J (2017). Interruption of the transmission of *Onchocerca volvulus* in the Kashoya-Kitomi focus, western Uganda by long-term ivermectin treatment and elimination of the vector *Simulium neavei* by larviciding. Acta Trop.

[31] Lakwo TL, Garms R, Tukahebwa E (2015). Successful interruption of the transmission of *Onchocerca volvulus* in Mpamba-Nkusi focus, Kibaale District, Uganda. East Afr Med J.

[32] Katabarwa M, Lakwo T, Habomugisha P (2014). Transmission of *Onchocerca volvulus* by *Simulium neavei* in Mount Elgon focus of eastern Uganda has been interrupted. Am J Trop Med Hyg.

[33] Katabarwa MN, Walsh F, Habomugisha P (2012). Transmission of onchocerciasis in Wadelai focus of northwestern Uganda has been interrupted and the disease eliminated. J Parasitol Res.

[34] Katabarwa MN, Habomugisha P, Khainza A (2020). Elimination of *Simulium neavei*-transmitted onchocerciasis in Wambabya-Rwamarongo focus of western Uganda. Am J Trop Med Hyg.

[35] Isiyaku S, Igbe M, Madaki S (2022). The interruption of transmission of onchocerciasis in Kaduna, Kebbi and Zamfara states, Nigeria: another milestone achievement. Int Health.

[36] Richards FO, Eigege A, Umaru J (2020). The interruption of transmission of human onchocerciasis by an annual mass drug administration program in Plateau and Nasarawa States, Nigeria. Am J Trop Med Hyg.

[37] Miri ES, Eigege A, Kahansim B (2022). Two Nigerian states (Plateau and Nasarawa) have eliminated transmission of human onchocerciasis—a report of post-ivermectin mass drug administration surveillance. Am J Trop Med Hyg.

[38] Higazi TB, Zarroug IMA, Mohamed HA (2013). Interruption of *Onchocerca volvulus* transmission in the Abu Hamed focus, Sudan. Am J Trop Med Hyg.

[39] Zarroug IM, Hashim K, ElMubark WA (2016). The first confirmed elimination of an onchocerciasis focus in Africa: Abu Hamed, Sudan. Am J Trop Med Hyg.

[40] Katabarwa MN, Zarroug IMA, Negussu N (2020). The Galabat-Metema cross-border onchocerciasis focus: the first coordinated interruption of onchocerciasis transmission in Africa. PLoS Negl Trop Dis.

[41] Diawara L, Traoré MO, Badji A (2009). Feasibility of onchocerciasis elimination with ivermectin treatment in endemic foci in Africa: first evidence from studies in Mali and Senegal. PLoS Negl Trop Dis.

[42] Katabarwa MN, Lakwo T, Habomugisha P (2018). After 70 years of fighting an age-old scourge, onchocerciasis in Uganda, the end is in sight. Int Health.

[43] Hougard JM, Alley ES, Yaméogo L, Dadzie KY, Boatin BA (2001). Eliminating onchocerciasis after 14 years of vector control: a proved strategy. J Infect Dis.

[44] Tekle AH, Elhassan E, Isiyaku S (2012). Impact of long-term treatment of onchocerciasis with ivermectin in Kaduna State, Nigeria: first evidence of the potential for elimination in the operational area of the African Programme for Onchocerciasis Control. Parasit Vectors.

[45] Muro AIS, Mziray NR (1990). Decline in onchocerciasis in the eastern Usambara mountains, north eastern Tanzania, and its possible relationship to deforestation. Acta Leiden.

[46] Muro AIS, Raybould JN (1990). Population decline of *Simulium woodi* and reduced onchocerciasis transmission at Amani, Tanzania, in relation to deforestation. Acta Leiden.

[47] Sauerbrey M, Rakers LJ, Richards FO (2018). Progress toward elimination of onchocerciasis in the Americas. Int Health.

[48] Lakwo T, Oguttu D, Ukety T, Post R, Bakajika D (2020). Onchocerciasis elimination: progress and challenges. Res Rep Trop Med.

[49] Cano J, Basáñez MG, O'Hanlon SJ (2018). Identifying co-endemic areas for major filarial infections in sub-Saharan Africa: seeking synergies and preventing severe adverse events during mass drug administration campaigns. Parasit Vectors.

[50] Koala L, Nikièma A, Post RJ (2017). Recrudescence of onchocerciasis in the Comoé valley in southwest Burkina Faso. Acta Trop.

[51] Koala L, Nikièma AS, Paré AB (2019). Entomological assessment of the transmission following recrudescence of onchocerciasis in the Comoé Valley, Burkina Faso. Parasit Vectors.

[52] Walker M, Stolk WA, Dixon MA (2017). Modelling the elimination of river blindness using long-term epidemiological and programmatic data from Mali and Senegal. Epidemics.

[53] Wilson NO, Badara Ly A, Cama VA (2016). Evaluation of lymphatic filariasis and onchocerciasis in three Senegalese districts treated for onchocerciasis with ivermectin. PLoS Negl Trop Dis.

[54] Koroma JB, Sesay S, Conteh A (2018). Impact of five annual rounds of mass drug administration with ivermectin on onchocerciasis in Sierra Leone. Infect Dis Poverty.

[55] Koudou BG, Kouakou MM, Ouattara AF (2018). Update on the current status of onchocerciasis in Côte d'Ivoire following 40 years of intervention: progress and challenges. PLoS Negl Trop Dis.

[56] Dolo H, Coulibaly YI, Sow M (2021). Serological evaluation of onchocerciasis and lymphatic filariasis elimination in the Bakoye and Falémé foci, Mali. Clin Infect Dis.

[57] Brieger WR, Okeibunor JC, Abiose AO (2007). Feasibility of measuring compliance to annual ivermectin treatment in the African Programme for Onchocerciasis Control. Trop Med Int Health.

[58] Brieger WR, Okeibunor JC, Abiose AO (2011). Compliance with eight years of annual ivermectin treatment of onchocerciasis in Cameroon and Nigeria. Parasit Vectors.

[59] Dyson L, Stolk WA, Farrell SH, Hollingsworth TD (2017). Measuring and modelling the effects of systematic non-adherence to mass drug administration. Epidemics.

[60] Newell ED (1997). Effect of mass treatments with ivermectin, with only partial compliance, on prevalence and intensity of *O. volvulus* infection in adults and in untreated 4 and 5 year-old children in Burundi. Trop Med Int Health.

[61] Wanji S, Kengne-Ouafo JA, Esum ME (2015). Relationship between oral declaration on adherence to ivermectin treatment and parasitological indicators of onchocerciasis in an area of persistent transmission despite a decade of mass drug administration in Cameroon. Parasit Vectors.

[62] Osue HO (2017). Field-based evidence of single and few doses of annual ivermectin treatment efficacy in eliminating skin microfilaria load after a decade of intervention. Ethiop J Health Sci.

[63] Jacob BG, Loum D, Lakwo TL (2018). Community-directed vector control to supplement mass drug distribution for onchocerciasis elimination in the Madi mid-North focus of Northern Uganda. PLoS Negl Trop Dis.

[64] Smith ME, Bilal S, Lakwo TL (2019). Accelerating river blindness elimination by supplementing MDA with a vegetation “slash and clear” vector control strategy: a data-driven modeling analysis. Sci Rep.

