# Supplementary material for: Elimination of transmission of onchocerciasis (river blindness) with long-term ivermectin mass drug administration with or without vector control in sub-Saharan Africa: a systematic review and meta-analysis
Source: Lancet Glob Health. 2024 Mar 11;12(5):e771–82. doi: 10.1016/S2214-109X(24)00043-3 (PMC11009120; doi:10.1016/S2214-109X(24)00043-3)
Supplement: Swahili translation of the abstract [file mmc1.pdf]

THE LANCET
Global Health

Supplementary appendix 1

This translation in Swahili was submitted by the authors and we reproduce it as supplied. It has not been peer reviewed. *The Lancet*'s editorial processes have only been applied to the original in English, which should serve as reference for this manuscript.

Tafsiri hii katika Swahili iliwasilishwa na waandishi na tunatengeneza tena kama hutolewa. Haijapitiwa. Mchakato wa hariri wa Lancet Global Health umetumika tu kwa asili kwa Kiingereza, ambayo inapaswa kutumika kama kumbukumbu kwa muswada hii.

Supplement to: Nyamai Mutono N, Basáñez M-G, James A, et al. Elimination of transmission of onchocerciasis (river blindness) with long-term ivermectin mass drug administration with or without vector control in sub-Saharan Africa: a systematic review and meta-analysis. *Lancet Glob Health* 2024; published online March 11. https://doi.org/10.1016/S2214-109X(24)00043-3.

Mukhtasari

Mandharinyuma: Shirika la Afya Duniani (WHO) limependekeza kutokomeza maambukizi ya onchocerciasis, kwa jina lingine River Blindness (upofu wa mto), ifikapo mwaka 2030. Zaidi ya asilimia tisaini na tisa ya visa vya ugonjwa huu vipo katika eneo la Afrika Kusini mwa Sahara. Kwa miaka mingi sasa, njia kuu za kutokomeza onchocerciais zimekuwa udhibiti wa wadudu na utoaji wa dawa ya ivermectin kwa wingi, ingawa mafanikio yamekuwa tofauti. Kwa utafiti huu, tulilenga kutambua sababu zinazohusiana na kumalizika kwa maambukizi ya onchocerciasis katika eneo la Afrika Kusini mwa Sahara.

Mbinu Tulifuata ukaguzi wa utaratibu pamoja na uchambuzi wa data kutoka karatasi za kisayansi zilizochapishwa kuhusu epidemiologia au tathmini ya wadudu wanohusiana na onchocerciasis katika eneo la Afrika Kusini mwa Sahara. Tulitafuta karatasi zilizokuwa kwa maktaba ya MEDLINE, PubMed, Web of Science, Embase, Cochrane Central Register of Controlled Trials, African Index Medicus, na Google Scholar kwa nakala zote zilizochapishwa kuanzia mwanzo wa kuhifadhi data hadi Agosti 19, 2023, bila kikwazo cha lugha. Ili kutafuta karatasi kwenye maktaba hizo, tulitumia maneno ya “onchocerciasis” NA “ivermectin” NA “utoaji wa dawa kwa wingi”. Tulitumia vigezo vitatu vya kujumuisha karatasi zilizochaguliwa: (1) kuhusiana na eneo la maambukizi lililokuwa Africa, (2) kuripoti kuhusu kutokomeza kwa maambukizi au angalau miaka 10 ya utoaji wa dawa ya ivermectin kwa wingi, na (3) kutumia angalau moja ya tathmini zifuatazo: kiwango cha microfilarial, kiwango cha uvimbe, kiwango cha Ov16 antibody, na kiwango cha kuambukiza kwa nzi wa kijivu. Karatasi za modeli ya hisibati au nakala za ukaguzi ziliondolewa. Waandishi wanne (NM, AJ, AM, na TNK) walichambua data kwenye nakala zilizochaguliwa na kuziweka kwenye Excel. Data za kiwango cha mtu binafsi hazikuombwa kutoka kwa waandishi. Maeneo yaligawanywa kwa mara tatu: yaliyofanikiwa kutokomeza maambukizi, yanayokaribia kutokomeza maambukizi, au yanayoendelea kuwa na maambukizi. Tulitumia mbinu za uchambuzi wa data za mixed-effects meta-regression models kutambua mambo yanayohusiana na hali ya maambukizi. Utafiti huu ulisajiliwa katika PROSPERO, CRD42022338986.

Matokeo: Baada ya kuodoa nakala zilizofanana, nakala 75 zilitimiza vigezo vya ujumuishi kwa kwa 1525 zilizochunguzwa. Nakala hizo zilitoa rekodi 282 kutoka kwa maeneo 238 tofauti, katika nchi 19 (70%) kwa 27 katika eneo la Afrika Kusini mwa Sahara zilizoathiriwa na onchocerciasis. Nakala 24 (9%) ziliripoti kutokomeza maambukizi, nakala 86 (30%) kukaribia kutokomeza maambukizi, na nakala 172 (61%) kuwa na maambukizi yakiendelea. I^2 ilikuwa 83.3% (95% CI 79.7 hadi 86.3). Rekodi zilizoripoti miaka 10 au zaidi ya kutumia ivermectin kwa wingi kufikia asilimia themanini au zaidi ya watu waliostahiki, zilikuwa na uwezekano zaidi wa kutokomeza maambukizi (log-odds 8.5 [95% CI 3.5 hadi 13.5]) au kutokomeza na kuwa karibu na kutokomeza maambukizi (42.4 [18.7 hadi 66.1]). Hii ni ikilinganishwa na rekondi ambazo hazikufikia asilimia themanini au zaidi ya matibabu. Kutokomeza au kuwa karibu na kutokomeza maambukizi kulihusishwa na zaidi na ripoti za miaka 15–19 za kupeana dawa za ivermectin kwa wingi kulinganishwa na matibabu chini ya miaka 15 (22.7 [17.2 hadi 28.2]), na pia matibabu mara mbili kwa mwaka kulinganishwa na matibabu yasiyofika mara mbili kwa mwaka (43.3 [27.2 hadi 59.3]). Hatari ya kuendelea kusambaa kwa ugonjwa ilikuwa juu kwa maeneo yaliyokuwa na kiwango cha juu cha ugonjwa (holoendemic) na pale udhibiti wa wadudu ulifanywa, yakilinganishwa na maeneo yaliyokuwa na kiwango cha chini cha ugonjwa (hypoendemic) na pale udhibiti wa wadudu haukutekelezwa. Kutoweka kwa nzi-weusi (blackflies) kutokana na udhibiti wa wadudu au mabadiliko ya mazingira kulisaidia kutokomeza maambukizi.

Tafsiri: Muda kwa miaka na mara ya utoaji wa dawa kwa wingi kwa mwaka, na asilimia ya watu waliopoekea matibabu; kiwango cha ugonjwa kwenye mwanzo, na kutokomeza kwa wadudu wabebaji ugonjwa kunahusiana na kutokomeza usambazaji wa onchocerciasis katika eneo la Afrika Kusini mwa Jangwa la Sahara. Matokeo yetu yanasisitiza umuhimu wa kuboresha na kudumisha matibabu kwa kiwango cha asilimia ya juu kwa watu wanaostahiki, na pia kuongeza mara ya kupatiana matibabu kwa mwaka ili kufanikiwa kutokomeza usambaji wa ugonjwa huu.

Ufadhili: Bill and Melinda Gates Foundation na Neglected Tropical Diseases Modelling Consortium, UK Medical Research Council, na Global Health EDCTp3 Joint Undertaking.
