# Supplementary material for: Elimination of transmission of onchocerciasis (river blindness) with long-term ivermectin mass drug administration with or without vector control in sub-Saharan Africa: a systematic review and meta-analysis
Source: Lancet Glob Health. 2024 Mar 11;12(5):e771–82. doi: 10.1016/S2214-109X(24)00043-3 (PMC11009120; doi:10.1016/S2214-109X(24)00043-3)
Supplement: French translation of the abstract [file mmc2.pdf]

THE LANCET
Global Health

Supplementary appendix 2

This translation in French was submitted by the authors and we reproduce it as supplied. It has not been peer reviewed. *The Lancet*'s editorial processes have only been applied to the original in English, which should serve as reference for this manuscript.

Cette traduction en français a été proposée par les auteurs et nous l'avons reproduite telle quelle. Elle n'a pas été examinée par des pairs. Les processus éditoriaux du *Lancet* n'ont été appliqués qu'à l'original en anglais et c'est cette version qui doit servir de référence pour ce manuscrit.

Supplement to: Nyamai Mutono N, Basáñez M-G, James A, et al. Elimination of transmission of onchocerciasis (river blindness) with long-term ivermectin mass drug administration with or without vector control in sub-Saharan Africa: a systematic review and meta-analysis. *Lancet Glob Health* 2024; published online March 11. https://doi.org/10.1016/S2214-109X(24)00043-3.

Résumé
Contexte L'OMS a proposé d'éliminer la transmission de l'onchocercose (cécité des rivières) d'ici 2030. Plus de 99 % des cas d'onchocercose surviennent en Afrique subsaharienne. La lutte anti- vectorielle et l'administration de masse des médicaments à base d'ivermectine sont les principales interventions mises en œuvre depuis de plusieurs années, avec un succès variable. L' objectif de notre étude était d'identifier les facteurs associés à l'élimination de l'onchocercose en Afrique subsaharienne.
Méthodes Pour cette revue systématique et méta-analyse, nous avons recherché des articles publiés faisant état d'évaluations épidémiologiques ou entomologiques de l'état de la transmission de l'onchocercose en Afrique subsaharienne, avec ou sans contrôle vectoriel. Nous avons effectué des recherches dans les bases de données MEDLINE, PubMed, Web of Science, Embase, Cochrane Central Register of Controlled Trials, African Index Medicus et Google Scholar pour tous les articles publiés depuis la création de chaque base de données jusqu'au 19 août 2023, sans restrictions de langue. Les termes de recherche utilisés étaient « onchocercose » ET « ivermectine » ET « administration de masse des médicaments ». Les trois critères d'inclusion étaient (1) le ou les foyer(s) situés en Afrique, (2) la déclaration d'une élimination de la transmission ou d'au moins 10 ans d'administration de masse de l'ivermectine dans le ou les foyer(s), et (3) l'inclusion d'au moins une des évaluations suivantes : prévalence des microfilaires , prévalence des nodules, séroprévalence des anticorps Ov16 et prévalence de l'infectiosité des simulies. Les études et revues de modélisation épidémiologique ont été exclues. Quatre évaluateurs (NM, AJ, AM et TNK) ont extrait les données en double exemplaire des articles en texte intégral à l'aide d'un outil d'extraction de données développé dans Excel avec des colonnes décrivant les données d'intérêt à extraire et une colonne contenant les commentaires importants pour chaque étudepourraientt être mis en avant. Nous n'avons demandé aucune donnée individuelle aux auteurs. Les foyers ont été classés comme ayant atteint l'élimination de la transmission, étant proches de l'élimination de la transmission ou avec une transmission continue. Nous avons utilisé des modèles de méta-régression à effets mixtes pour identifier les facteurs associés au statut de transmission. Cette étude est enregistrée dans PROSPERO, CRD42022338986.
Résultats Sur 1 525 articles examinés après suppression des doublons, 75 ont fourni 282 enregistrements provenant de 238 foyers distincts dans 19 (70 %) des 27 pays d'endémie de l'onchocercose en Afrique subsaharienne. L'élimination de la transmission a été observée dans 24 (9 %) enregistrements, l'élimination de la transmission était proche dans 86 (30 %) enregistrements et 172 (61%) enregistrements avaient une transmission continue. I^2 était de 83,3 % (IC à 95 % 79,7 à 86,3). Les enregistrements faisant état de 10 années ou plus d'administration de massecontinue de médicaments avec une couverture thérapeutique de 80 % ou plus de la population éligible avaient des chances significativement plus élevées d'éliminer la transmission (log-cotes 8,5[IC à 95 % 3,5 à 13,5]) oud'atteindre l' élimination ou la quasi-élimination de la transmission (42,4 [18,7 à 66,1]) comparé à ceux qui n'en ont pas. Les rapports sur 15 à 19 ans d'administration massive de l'ivermectine (22,7 [17,2 à 28,2]) et de traitement semestriel (43,3 [27,2 à 59,3]) étaient positivement associés à l'élimination ou à la quasi-élimination. de la transmission, comparé aux rapports sur moins de 15 ans et aucune administration massive de médicaments semestrielle, respectivement. Le fait d'avoir subi un contrôle vectoriel (–42,8 [–59,1 à –26,5]) et une holoendémicité initiale (–41,97 [–60,6 à –23,2]) étaient associés à un risque accru de transmission continue par rappcomparéort à l'absence de lutte anti-vectorielle et à l'hypoendémicité , respectivement. La disparition des simulies due à la lutte anti-vectorielle ou aux changements environnementaux a contribué à l'élimination de la transmission.
Interprétation : La durée, fréquence et couverture de l'administration de masse de médicaments ; l'endémicité de base ; et l'élimination ou la disparition des populations vectorielles sont des déterminants importants de l'élimination de la transmission de l'onchocercose en Afrique sub-saharienne. Nos résultats soulignent l'importance d'améliorer et de maintenir la couverture thérapeutique pour que les pays parviennent à l'élimination de la transmission de l'onchocercose.
Financement Fondation Bill & Melinda Gates, Consortium de modélisation des maladies tropicales négligées, Conseil de la recherche médicale du Royaume-Uni, et Global Health EDCTP3 Joint Undertaking.
