# Supplementary material for: Elimination of transmission of onchocerciasis (river blindness) with long-term ivermectin mass drug administration with or without vector control in sub-Saharan Africa: a systematic review and meta-analysis
Source: Lancet Glob Health. 2024 Mar 11;12(5):e771–82. doi: 10.1016/S2214-109X(24)00043-3 (PMC11009120; doi:10.1016/S2214-109X(24)00043-3)
Supplement: Spanish translation of the abstract [file mmc3.pdf]

THE LANCET
Global Health

Supplementary appendix 3

This translation in Spanish was submitted by the authors and we reproduce it as supplied. It has not been peer reviewed. *The Lancet*'s editorial processes have only been applied to the original in English, which should serve as reference for this manuscript.

Los autores nos proporcionaron esta traducción al español y la reproducimos tal como nos fue entregada. No la hemos revisado. Los procesos editoriales de *The Lancet* se han aplicado únicamente al original en inglés, que debe servir de referencia para este manuscrito.

Supplement to: Nyamai Mutono N, Basáñez M-G, James A, et al. Elimination of transmission of onchocerciasis (river blindness) with long-term ivermectin mass drug administration with or without vector control in sub-Saharan Africa: a systematic review and meta-analysis. *Lancet Glob Health* 2024; published online March 11. https://doi.org/10.1016/S2214-109X(24)00043-3.

Resumen

Antecedentes. La Organización Mundial de la Salud (OMS) ha propuesto metas para la eliminación de la transmisión de la oncocercosis (ceguera de los ríos) para el año 2030. Al menos el 99% de los casos de la enfermedad se encuentran en África sub-sahariana. Las principales medidas de intervención se han basado en el control de vectores y la administración masiva de ivermectina por muchos años, con éxito variable. Nuestro objetivo es el de identificar factores asociados con la eliminación de la transmisión de la oncocercosis en África sub-sahariana.

Métodos. Para esta revisión sistemática y meta-análisis se realizó una búsqueda de artículos publicados que han reportado resultados de evaluaciones epidemiológicas y entomológicas sobre el estado de la transmisión de la oncocercosis en África sub-sahariana, con o sin control vectorial. Las bases de datos utilizadas fueron las de MEDLINE, PubMed, Web de la Ciencia, Embase, Registro Cochrane Central de Ensayos Controlados, Index Medicus Africano y Google Académico, desde la incepción de las mismas hasta el 19 de agosto del 2023, sin restricción de idiomas. Los términos utilizados fueron “oncocercosis” E “ivermectina” Y “administración masiva de medicamentos”. Los tres criterios de inclusión fueron (1) foco o focos de oncocercosis localizados en África; (2) reportando eliminación o al menos 10 años de administración masiva de ivermectina, y (3) inclusión de al menos uno de los siguientes resultados: prevalencia de microfilarias, prevalencia de nódulos oncocercóticos, seroprevalencia de anticuerpos contra el antígeno Ov16, y prevalencia de infectividad en los simúlidos vectores. Estudios de modelaje matemático y de revisión fueron excluidos. Cuatro revisores (NM, AJ, AM, y TNK) obtuvieron los datos en duplicado del texto completo de los artículos utilizando un instrumento de extracción desarrollado en Excel, con columnas describiendo los datos de interés a ser extraídos y una columna adicional a fin de resaltar comentarios importantes para cada estudio. No se solicitaron datos individuales a los autores de los estudios. Los focos identificados fueron clasificados como: aquellos alcanzando la eliminación de la transmisión, aquellos que están cercanos a la eliminación, y aquellos en los cuales la transmisión aún continúa. Modelos de meta-regresión con efectos mixtos fueron utilizados a fin de identificar factores asociados con la situación de la transmisión. El estudio fue registrado en PROSPERO, CRD42022338986.

Hallazgos. De los 1525 artículos tamizados luego de la remoción de duplicados, 75 proporcionaron 282 registros provenientes de 238 focos distintos en 19 (70%) de los 27 países actualmente endémicos en África sub-sahariana. Veinticuatro (9%) de los registros reportaron eliminación de la transmisión, 86 (30%) reportaron estar cercanos a la eliminación, y 172 (61%) que la transmisión continúa. El valor de I^2 fue del 83·3% (IC 95% 79·7 a 86·3). El reportar al menos 10 años continuos de administración masiva de ivermectina con un mínimo de cobertura terapéutica del 80% de población elegible resultó en logaritmos de la razón de probabilidades significativamente más altos de alcanzar la eliminación de la transmisión (8·5 [IC 95% 3·5 a 13·5]) o eliminación de la transmisión y estar cerca de la eliminación de la transmisión (42·4 [18·7 a 66·1]) que el no haber reportado dichas condiciones. La administración masiva de ivermectina por 15–19 años (22·7 [17·2 a 28·2]), y el tratamiento bianual (43·3 [27·2 a 59·3]) demostraron una asociación positiva con la eliminación de la transmisión y estar cerca de la eliminación en comparación con duraciones de tratamiento por debajo de 15 años o sin administración bianual, respectivamente. El haber implementado medidas anti-vectoriales (−42·8 [−59·1 a −26·5]) o haber tenido niveles holoendémicos basales (−41·97 [−60·6 a −23·2] estuvieron asociados con un mayor riesgo de presentar transmisión continua en comparación con no haber tenido control de vectores o con niveles basales de hipoendemicidad, respectivamente. La desaparición de los simúlidos debido al control vectorial o al cambio ambiental contribuyó a la eliminación de la transmisión.

Interpretación. La duración, frecuencia y cobertura de la administración masiva de ivermectina, así como los niveles basales de endemicidad y la eliminación o desaparición de los vectores constituyen importantes determinantes de la eliminación de la transmisión de la oncocercosis en África sub-sahariana. Estos hallazgos resaltan la importancia de mejorar y sostener altas coberturas terapéuticas e incrementar la frecuencia de tratamiento a fin de que los países endémicos puedan alcanzar la eliminación de la transmisión de la oncocercosis.

Financiamiento. La Fundación Bill & Melinda Gates, el Consorcio de Modelaje de las Enfermedades Tropicales Desatendidas, y el Concejo de Investigaciones Médicas del Reino Unido en empresa conjunta con el proyecto de Salud Global del EDCTP3.
