# Supplementary material for: Elimination of transmission of onchocerciasis (river blindness) with long-term ivermectin mass drug administration with or without vector control in sub-Saharan Africa: a systematic review and meta-analysis
Source: Lancet Glob Health. 2024 Mar 11;12(5):e771–82. doi: 10.1016/S2214-109X(24)00043-3 (PMC11009120; doi:10.1016/S2214-109X(24)00043-3)
Supplement: Portuguese translation of the abstract [file mmc4.pdf]

THE LANCET
Global Health

Supplementary appendix 4

This translation in Portuguese was submitted by the authors and we reproduce it as supplied. It has not been peer reviewed. *The Lancet*'s editorial processes have only been applied to the original in English, which should serve as reference for this manuscript.

Esta tradução em português foi submetida pelos autores e nós não fizemos quaisquer alterações. Esta versão não foi revista por pares. O processo editorial do The Lancet só foi aplicado à versão original em inglês, que deve servir como referência para este artigo.

Supplement to: Nyamai Mutono N, Basáñez M-G, James A, et al. Elimination of transmission of onchocerciasis (river blindness) with long-term ivermectin mass drug administration with or without vector control in sub-Saharan Africa: a systematic review and meta-analysis. *Lancet Glob Health* 2024; published online March 11. https://doi.org/10.1016/S2214-109X(24)00043-3.

Sumário

Introdução: A Organização Mundial de Saúde (OMS) propôs a eliminação da transmissão da oncocercose (cegueira dos rios) até 2030. Mais de 99% dos casos de oncocercose ocorrem na África Subsariana. O controlo vetorial e a administração em massa de ivermectina têm sido as principais intervenções há muitos anos, com sucesso variável. Este estudo tem como objetivo identificar os fatores associados com a eliminação da transmissão da oncocercose na África Subsaariana.

Métodos: Para esta revisão sistemática e meta-análise, procurámos artigos publicados relacionados com avaliações epidemiológicas ou entomológicas do estado de transmissão da oncocercose na África Subsariana, com ou sem controlo vetorial. Pesquisamos nas bases de dados MEDLINE, PubMed, Web of Science, Embase, Cochrane Central Register of Controlled Trials, African Index Medicus e Google Scholar todos os artigos publicados desde o início da base de dados até 19 de agosto de 2023, sem restrições de idioma. Os termos de pesquisa utilizados foram "onchocerciasis" AND "ivermectin" AND "mass drug administration".

Os três critérios de inclusão foram (1) foco ou focos localizados em África, (2) notificação de eliminação da transmissão ou pelo menos 10 anos de administração em massa de medicamentos de ivermectina no foco ou focos, e (3) inclusão de pelo menos uma das seguintes avaliações: prevalência de microfilárias, prevalência de nódulos, seroprevalência de anticorpos Ov16 e prevalência de infecciosidade em moscas negras. Foram excluídos estudos de modelagem epidemiológica e artigos de revisão. Quatro revisores (NM, AJ, AM e TNK) extraíram os dados em duplicado dos textos completos dos artigos usando uma ferramenta de extração de dados desenvolvida em Excel com colunas representando os dados de interesse a serem extraídos e uma coluna destacando comentários importantes para cada estudo. Nenhum dado de nível individual foi solicitado a autores.

Os focos foram classificados como alcançando a eliminação da transmissão, estando próximos da eliminação da transmissão ou com transmissão contínua. Utilizamos modelos de meta-regressão de efeitos mistos para identificar fatores associados ao estadio de transmissão. Este estudo está registrado no PROSPERO, CRD42022338986.

Resultados: Dos 1525 artigos analisados após a remoção de duplicados, 75 forneceram 282 registros de 238 focos distintos em 19 (70%) dos 27 países endêmicos de oncocercose na África Subsaariana. A eliminação da transmissão foi relatada em 24 (9%) registros, com 86 (30%) registros estando próximos da eliminação da transmissão, e 172 (61%) registros de transmissão contínua. I^2 foi de 83,3% (IC 95% 79,7 a 86,3). Registros com relatos de 10 ou mais anos de administração contínua de ivermectina em massa com 80% ou mais de cobertura terapêutica da população elegível demonstraram logaritmos da proporção de probabilidades significativamente maiores de alcançar a eliminação da transmissão (chances logarítmicas 8,5 [IC 95% 3,5 a 13,5]) ou eliminação e estando próximos da eliminação da transmissão (42,4 [18,7 a 66,1]) do que aqueles sem relatos semelhantes. Registros com 15–19 anos de administração em massa de ivermectina (22,7 [17,2 a 28,2]) e tratamento bianual (43,3 [27,2 a 59,3] foram positivamente associados à eliminação e estando proximos da eliminação de transmissão em comparação com menos de 15 anos e nenhuma administração bianual em massa de ivermectina, respectivamente. Ter tido controlo vetorial (−42,8 [−59,1 a

−26,5]) e holo-endemicidade de base (−41,97 [−60,6] a -23·2]) foi associado ao aumento do risco de transmissão contínua em comparação com nenhum controlo vetorial e hipo-endemicidade, respectivamente. O desaparecimento da mosca negra devido ao controlo vetorial ou mudanças ambientais contribuiu para a eliminação da transmissão.

Interpretação: A duração, frequência e cobertura da administração massiva de ivermectina; endemicidade de base; e a eliminação ou desaparecimento do vector são determinantes importantes da eliminação da transmissão da oncocercose na África Subsariana. Os nossos resultados sublinham a importância de melhorar e manter alta cobertura terapêutica e aumentar a frequência do tratamento para que os países consigam eliminar a transmissão da oncocercose.

Financiamento: Este estudo foi financiado pela Fundação Bill & Melinda Gates e o Consórcio de Modelagem Matemática de Doenças Tropicais Negligenciadas, Conselho de Investigação Médica do Reino Unido juntamente com o projeto Global Health EDCTP3.
