# Supplementary material for: Elimination of transmission of onchocerciasis (river blindness) with long-term ivermectin mass drug administration with or without vector control in sub-Saharan Africa: a systematic review and meta-analysis
Source: Lancet Glob Health. 2024 Mar 11;12(5):e771–82. doi: 10.1016/S2214-109X(24)00043-3 (PMC11009120; doi:10.1016/S2214-109X(24)00043-3)
Supplement: Supplementary appendix 5 [file mmc5.pdf]

THE LANCET
Global Health

Supplementary appendix 5

This appendix formed part of the original submission and has been peer reviewed. We post it as supplied by the authors.

Supplement to: Nyamai Mutono N, Basáñez M-G, James A, et al. Elimination of transmission of onchocerciasis (river blindness) with long-term ivermectin mass drug administration with or without vector control in sub-Saharan Africa: a systematic review and meta-analysis. *Lancet Glob Health* 2024; published online March 11. https://doi.org/10.1016/S2214-109X(24)00043-3.

Supplementary appendix

This appendix forms part of the original submission and will be peer reviewed.

Supplement to:

Elimination of transmission of onchocerciasis (river blindness) with long-term ivermectin mass drug administration with or without vector control in sub-Saharan Africa: a systematic review and meta-analysis

Nyamai Mutono PhD[1,2*], Prof. Maria-Gloria Basáñez PhD[3*], Ananthu James PhD[4], Wilma A. Stolk PhD[4], Anita Makori BSc[1,2], Teresia Njoki Kimani MSc[1,2,5], Prof. T. Déirdre Hollingsworth PhD[6], Andreia Vasconcelos PhD[6], Matthew A. Dixon PhD[3], Prof. Sake J. de Vlas PhD[4†], Prof. S.M. Thumbi PhD[1,2,7†]

[1] Centre for Epidemiological Modelling and Analysis, University of Nairobi, Nairobi, Kenya

[2] Paul G Allen School for Global Health, Washington State University, WA, USA

[3] MRC Centre for Global Infectious Disease Analysis (MRC GIDA) and London Centre for Neglected Tropical Disease Research (LCNTDR), School of Public Health, Department of Infectious Disease Epidemiology, Imperial College London, London, UK

[4] Department of Public Health, Erasmus MC, University Medical Center Rotterdam, Rotterdam, The Netherlands

[5] Ministry of Health Kenya, Kiambu County, Kenya

[6] Big Data Institute, University of Oxford, Oxford, UK

[7] Institute of Immunology and Infection Research, University of Edinburgh, Edinburgh, UK

* Co-first authors
[†] Contributed equally

Correspondence to: Mutono Nyamai, Centre for Epidemiological Modelling and Analysis, University of Nairobi, P.O Box 30197-00100, Nairobi Kenya
mutono.nyamai@uonbi.ac.ke

Search strategy used in each database

Onchocerciasis, Onchocerca volvulus
AND
ivermectin, mectizan
AND
mass drug administration, community directed treatment
AND
prevalence, microfilarial, microfilariae, microfilaria, endemicity, biting rate, microfilarial load, Ov16, elimin [ation] [ated], interruption, L3

Table S1: Detailed search strategy and records retrieved from each database

Database searched	Platform	Search strategy	Records	Records after duplicates removed
Embase	Embase.com	('onchocerciasis'/exp OR 'Onchocerca volvulus'/de OR (Onchocerc* OR onchodermat* OR river-blindness*):ab,ti,kw) AND ('ivermectin'/de OR (ivermectin* OR mectizan*):ab,ti,kw) AND ('mass drug administration'/de OR 'group therapy'/de OR 'epidemiology'/de OR 'mass immunization'/de OR 'parasite transmission'/de OR (mass OR ((communit*) NEAR/3 (administrat* OR treatment* OR immunization* OR immunisation* OR distribution* OR therap* OR chemotherap* OR vaccination*)) OR epidemiolog* OR ((parasite* OR elimination) NEAR/3 (transmission* OR public-health-problem*)) OR EoT OR EPHP):ab,ti,kw) NOT (([Conference Abstract]/lim OR [Conference Review]/lim)	1099	1077
MEDLINE	Ovid	(exp Onchocerciasis/ OR Onchocerca volvulus/ OR (Onchocerc* OR onchodermat* OR river-blindness*).ab,ti,kf.) AND (Ivermectin/ OR (ivermectin* OR mectizan*).ab,ti,kf.) AND (Mass Drug Administration/ OR Epidemiology/ OR exp Onchocerciasis/ep OR Onchocerca volvulus/ep OR Mass Vaccination/ OR Disease Transmission, Infectious/ OR (mass OR ((communit*) ADJ3 (administrat* OR treatment* OR immunization* OR immunisation* OR distribution* OR therap* OR chemotherap* OR vaccination*)) OR epidemiolog* OR ((parasite* OR elimination) ADJ3 (transmission* OR public-health-problem*)) OR EoT OR EPHP).ab,ti,kf.) NOT ((news OR congres* OR abstract* OR book* OR chapter* OR dissertation abstract*).pt.)	922	182
Web of Science* Core Collection	Web of Knowledge	TS=(((Onchocerc* OR onchodermat* OR river-blindness*)) AND ((ivermectin* OR mectizan*)) AND ((mass OR ((communit*) NEAR/2 (administrat* OR treatment* OR immunization* OR immunisation* OR distribution* OR therap* OR chemotherap* OR vaccination*)) OR epidemiolog* OR ((parasite* OR elimination) NEAR/2 (transmission* OR public-health-problem*)) OR EoT OR EPHP))) AND DT=(Article OR Review OR Letter OR Early Access)	840	181
Cochrane Central Register of Controlled Trials**	Wiley	((Onchocerc* OR onchodermat* OR river-blindness*):ab,ti) AND ((ivermectin* OR mectizan*):ab,ti) AND ((mass OR ((communit*) NEAR/3 (administrat* OR treatment* OR immunization* OR immunisation* OR distribution* OR therap* OR chemotherap* OR vaccination*)) OR epidemiolog* OR ((parasite* OR elimination) NEAR/3 (transmission* OR public-health-problem*)) OR EoT OR EPHP):ab,ti) NOT "conference abstract":pt	37	1
African Index Medicus (WHO)	https://www.globalindexmedicus.net/	TW:((Onchocerc* OR onchodermat* OR river-blindness*) AND (ivermectin* OR mectizan*) AND (mass OR community OR epidemiolog* OR EoT OR EPHP))	24	16

Google Scholar (200 top ranked)		onchocerciasis|"river-blindness" ivermectin|mectizan "mass|community administration|treatment|immunization|immunisation|distribution|therapy|chemotherapy|vaccination" onchocerciasis|'river-blindness' ivermectin|mectizan 'mass|community administration|treatment|immunization|immunisation|distribution|therapy|chemotherapy|vaccination'	220	68
**Total**				**1525**

*Science Citation Index Expanded (1975–present); Social Sciences Citation Index (1975–present); Arts & Humanities Citation Index (1975–present); Conference Proceedings Citation Index–Science (1990–present); Conference Proceedings Citation Index–Social Science & Humanities (1990–present); Emerging Sources Citation Index (2005–present).
** Manually deleted abstracts from trial registries

Table S2: Criteria used for abstract screening

Criterion	Guidance	Outcome
Study foci	Does the Title/Abstract exclusively target foci or endemic areas outside the African region?	If yes, exclude<br><br>If no, include
Treatment	Does the Title/Abstract mention use of ivermectin or Mectizan® as a therapeutic treatment for onchocerciasis for at least 10 years?	If yes, include<br><br>If no, exclude
Endemicity	Does the Title/Abstract mention endemicity either through microfilarial prevalence, palpable nodule prevalence or microfilarial load in the study population?	If yes, include<br><br>If no, exclude

Table S3: Variables extracted

Data extracted	Description
Authors and Year of publication	
Journal title and Article title	
Country of focus	Country or countries where the study took place
Subnational area	States, districts, foci or villages where the study was conducted
Year data were collected	When the study was conducted
Population sample size	Study population and sample size for each study foci
Ecological features	Savannah, forest, or forest-savannah mosaic
Vector details	*Simulium* species, baseline annual biting rate (no. bites/person/year) and annual transmission potential (no. L3 larvae/person/year)
Baseline (pre-intervention) endemicity	Level of onchocerciasis endemicity at baseline (from hypo- to holoendemicity) according to microfilarial prevalence, onchocercal nodule prevalence, and/or community microfilarial load (CMFL)
Vector control	Presence or absence of vector control; number of years of vector control; type of vector control (aerial or ground larviciding of vector breeding sites)
Ivermectin MDA treatment	Number of years of ivermectin treatment in the study area; geographical coverage; therapeutic coverage of eligible population (if ≥ 80%, record the number of years it has been above this level); treatment frequency (yearly or more frequently; if shift to biannual or quarterly, record the number of years of change in frequency); proportion of the population adhering or not to treatment; proportion never treated.
Microfilarial prevalence and CMFL	Methods for collecting and incubating skin snips, detecting and enumerating microfilariae; calculation of microfilarial prevalence and CMFL, age groups and sample sizes
Nodule prevalence	Method for recording palpable nodules; age groups and sample sizes
Diagnostic tests used in Stop-MDA surveys	Children samples, ages and sample size; method for IgG4 antibody testing (ELISA, RDT); blackfly samples, number of flies tested and method for recording fly infection/infectivity. Ov16 seroprevalence (with 95% CIs), fly infectivity rate (with 95% CIs)
Co-endemicity	Record co-endemic infections (*Loa loa*, *Wuchereria bancrofti*)
Other important data collected	Record other clinical manifestations; duration of civil conflict; MDA interruption

Table S4: Quality assessment of the 75 studies using the STROBE and NIH checklists

Author and year of publication	Does the study have a clearly stated objective?	Is there a clear study design?	Is there a description of the study location?	Are there dates of data collection?	Are there participant eligibility criteria clearly stated?	Have the vector species been identified?	Has the morbidity examination clearly been described?	Has the parasito-logical examination been clearly described?	Is there a discussion of study limitations?	Have sources of funding been mentioned?	Quality
Abong et al., 2020[1]	Yes	Yes	Yes	Yes	Yes	No	Yes	Yes	Yes	Yes	High
Anosike et al., 2007[2]	Yes	Yes	Yes	Yes	Yes	No	Yes	–	No	No	High
Aza'ah et al., 2020[3]	Yes	Yes	Yes	Yes	Yes	No	Yes	–	No	Yes	High
Bakajika et al., 2018[4]	Yes	Yes	Yes	Yes	Yes	Yes	Yes	Yes	No	Yes	High
Biritwum et al., 2021[5]	Yes	Yes	No	Yes	Yes	No	Yes	Yes	No	Yes	High
Borsboom et al., 2003[6]	Yes	Yes	Yes	Yes	Yes	Yes	No	Yes	No	No	High
Diawara et al., 2009[7]	Yes	Yes	Yes	Yes	Yes	Yes	Yes	Yes	No	Yes	High
Djune-Yemeli et al., 2022[8]	Yes	Yes	Yes	Yes	Yes	No	Yes	No	Yes	Yes	High
Dolo et al., 2021[9]	Yes	Yes	Yes	Yes	Yes	Yes	Yes	–	No	Yes	High
Evans et al., 2014[10]	Yes	Yes	Yes	Yes	Yes	Yes	Yes	Yes	Yes	Yes	High
Forrer et al., 2021[11]	Yes	Yes	Yes	Yes	Yes	No	Yes	–	Yes	Yes	High
Garms et al., 2015[12]	Yes	Yes	Yes	Yes	No	Yes	–	Yes	No	Yes	High
Gebrezgabiher et al., 2020[13]	Yes	Yes	Yes	Yes	Yes	No	Yes	Yes	Yes	Yes	High
Hendy et al., 2018[14]	Yes	Yes	Yes	Yes	Yes	Yes	–	Yes	Yes	Yes	High
Hernández-González et al., 2016[15]	Yes	Yes	Yes	Yes	Yes	Yes	Yes	Yes	Yes	Yes	High
Herrador et al., 2018[16]	Yes	Yes	Yes	Yes	Yes	Yes	Yes	Yes	Yes	Yes	High
Higazi et al., 2013[17]	Yes	Yes	Yes	Yes	Yes	Yes	Yes	Yes	No	Yes	High
Johanns et al., 2022[18]	Yes	Yes	Yes	Yes	Yes	Yes	Yes	Yes	No	Yes	High
Kamga et al., 2016[19]	Yes	Yes	Yes	Yes	Yes	No	Yes	No	No	Yes	Moderate
Kamga et al., 2017[20]	Yes	Yes	Yes	Yes	Yes	No	Yes	No	No	Yes	Moderate
Kamga et al., 2018[21]	Yes	Yes	Yes	Yes	Yes	No	No	–	Yes	Yes	High
Katabarwa et al., 2008[22]	Yes	Yes	Yes	Yes	Yes	No	Yes	Yes	No	Yes	High
Katabarwa et al., 2011[23]	Yes	Yes	Yes	Yes	Yes	Yes	Yes	Yes	No	Yes	High
Katabarwa et al., 2012[24]	Yes	Yes	Yes	Yes	Yes	Yes	Yes	Yes	No	Yes	High

Katabarwa et al., 2013a[25]	Yes	Yes	Yes	Yes	Yes	Yes	Yes	Yes	No	Yes	High
Katabarwa et al., 2013b[26]	Yes	Yes	Yes	Yes	Yes	Yes	Yes	Yes	No	No	High
Katabarwa et al., 2014[27]	Yes	Yes	Yes	Yes	No	Yes	Yes	–	No	Yes	High
Katabarwa et al., 2016[28]	Yes	Yes	Yes	Yes	Yes	Yes	Yes	No	No	No	High
Katabarwa et al., 2020a[29]	Yes	Yes	Yes	Yes	Yes	Yes	Yes	Yes	No	Yes	High
Katabarwa et al., 2020b[30]	Yes	Yes	Yes	Yes	Yes	Yes	Yes	Yes	No	Yes	High
Kogi & Bulus, 2008[31]	Yes	Yes	Yes	Yes	No	No	No	No	No	No	Moderate
Komlan et al., 2018[32]	Yes	Yes	Yes	No	Yes	Yes	Yes	Yes	No	Yes	High
Koudou et al., 2018[33]	Yes	Yes	Yes	Yes	Yes	Yes	Yes	–	Yes	Yes	High
Lakwo et al., 2013[34]	Yes	Yes	Yes	Yes	Yes	Yes	Yes	–	No	Yes	High
Lakwo et al., 2015[35]	Yes	Yes	Yes	Yes	Yes	Yes	Yes	–	Yes	Yes	High
Lakwo et al., 2017[36]	Yes	Yes	Yes	Yes	Yes	Yes	Yes	No	No	Yes	High
Lakwo Luroni et al., 2017[37]	Yes	Yes	Yes	Yes	Yes	Yes	Yes	Yes	No	Yes	High
Lamberton et al., 2015[38]	Yes	Yes	Yes	Yes	Yes	Yes	–	Yes	Yes	Yes	High
Moya et al., 2016[39]	Yes	Yes	Yes	Yes	Yes	Yes	Yes	No	Yes	Yes	High
Mweya et al., 2007[40]	Yes	Yes	Yes	Yes	Yes	Yes	Yes	–	No	Yes	High
Nyagang et al., 2020[41]	Yes	Yes	Yes	Yes	Yes	No	Yes	–	Yes	No	High
Okon & Ilugbiyin, 2008[42]	Yes	Yes	Yes	Yes	Yes	Yes	Yes	Yes	No	No	High
Osei-Atweneboana et al., 2007[43]	Yes	Yes	Yes	Yes	Yes	Yes	Yes	Yes	Yes	Yes	High
Osue et al., 2013[44]	Yes	Yes	Yes	Yes	No	Yes	Yes	–	No	Yes	High
Osue, 2017[45]	Yes	Yes	Yes	Yes	Yes	No	Yes	No	No	Yes	High
Otabil et al., 2019[46]	Yes	Yes	Yes	Yes	Yes	No	Yes	No	No	No	Moderate
Paulin et al., 2017[47]	Yes	Yes	Yes	Yes	Yes	No	No	–	No	Yes	High
Richards et al., 2020[48]	Yes	Yes	Yes	Yes	Yes	Yes	Yes	Yes	No	Yes	High
Sam-Wobo et al., 2012[49]	Yes	Yes	Yes	Yes	Yes	Yes	Yes	–	No	Yes	High
Seidenfaden et al., 2001[50]	Yes	Yes	Yes	Yes	Yes	Yes	Yes	Yes	No	Yes	High
Shintouo et al., 2020[51]	Yes	Yes	Yes	Yes	Yes	Yes	Yes	Yes	Yes	Yes	High
Siewe Fodjo et al., 2019[52]	Yes	Yes	Yes	Yes	Yes	Yes	Yes	–	Yes	Yes	High
Sufi & Zainab, 2015[53]	Yes	Yes	Yes	Yes	Yes	No	No	–	No	No	Moderate
Surakat et al., 2018[54]	Yes	Yes	Yes	Yes	Yes	Yes	Yes	–	No	Yes	High

Ta et al., 2018[55]	Yes	Yes	Yes	Yes	Yes	No	Yes	–	No	Yes	High
Tekle et al., 2012[56]	Yes	Yes	Yes	Yes	Yes	Yes	Yes	Yes	Yes	Yes	High
Tekle et al., 2016[57]	Yes	Yes	Yes	Yes	Yes	Yes	Yes	–	No	Yes	High
Traoré et al., 2009[58]	Yes	Yes	Yes	Yes	Yes	Yes	Yes	–	No	No	High
Traore et al., 2012[59]	Yes	Yes	Yes	Yes	Yes	Yes	Yes	Yes	No	Yes	High
Wanji et al., 2015[60]	Yes	Yes	Yes	Yes	Yes	Yes	Yes	Yes	No	Yes	High
Wilson et al., 2016[61]	Yes	Yes	Yes	Yes	Yes	Yes	Yes	Yes	Yes	Yes	High
Yaya et al., 2014[62]	Yes	Yes	Yes	Yes	Yes	Yes	Yes	Yes	Yes	No	High
Zarroug et al., 2016[63]	Yes	Yes	Yes	Yes	Yes	Yes	Yes	Yes	No	Yes	High
Mshana et al., 2023[64]	Yes	Yes	Yes	Yes	Yes	Yes	Yes	–	Yes	Yes	High
Atekem et al., 2022[65]	Yes	Yes	Yes	Yes	Yes	Yes	Yes	Yes	Yes	Yes	High
Otabil et al., 2023[66]	Yes	Yes	Yes	Yes	Yes	Yes	Yes	–	Yes	Yes	High
Chikezie et al., 2023[67]	Yes	Yes	Yes	No	Yes	Yes	Yes	Yes	Yes	Yes	High
Ekpo et al., 2022[68]	Yes	Yes	Yes	Yes	Yes	No	Yes	–	Yes	Yes	High
Bhwana et al., 2023[69]	Yes	Yes	Yes	Yes	Yes	Yes	Yes	–	Yes	Yes	High
Efon-Ekanguouo et al., 2023[70]	Yes	Yes	Yes	Yes	Yes	No	Yes	–	Yes	Yes	High
Miri et al., 2023[71]	Yes	Yes	Yes	Yes	Yes	Yes	Yes	Yes	Yes	Yes	High
Anagbogu et al., 2022[72]	Yes	Yes	Yes	Yes	Yes	No	Yes	–	Yes	Yes	High
Ekanya et al., 2022[73]	Yes	Yes	Yes	Yes	Yes	Yes	Yes	Yes	Yes	Yes	High
Domche et al., 2022[74]	Yes	Yes	Yes	Yes	Yes	No	Yes	Yes	Yes	Yes	High
Isiyaku et al., 2022[75]	Yes	Yes	Yes	Yes	Yes	Yes	Yes	Yes	Yes	Yes	High

Table S5: I^2 and τ^2 values for quantifying heterogeneity across studies

Categories	Sub-categories	Heterogeneity statistic	Degrees of freedom	I^2	τ^2
Region	Central Africa (Ref)				
	East Africa	16643.5	282	99.63%	0.02
	West Africa				
Ecological features	Savannah (Ref)				
	Forest	6704.7	169	99.98%	0.02
	Forest-savannah mosaic				
Vector control (Larviciding)	No (Ref)				
	Yes	16786.5	282	98.82%	0.02
Number of years of ivermectin treatment		14458.1	282	99.65%	0.01
Proportion never treated	≤5% (Ref)				
	>5%	3067.2	30	99.95%	0.02
Years of continuous therapeutic treatment at ≥80% coverage of the eligible population	<10 years				
	≥10 years	16658.7	195	99.35%	0.02
Baseline endemicity	Hypoendemicity (Ref)				
	Mesoendemicity	16647.4	282	99.79%	0.02
	Hyperendemicity				
	Holoendemicity				
Co-endemicity	Not reported				
	Loa loa	16285.2	282	99.91%	0.02
	Wuchereria bancrofti				

Ref: Reference group.

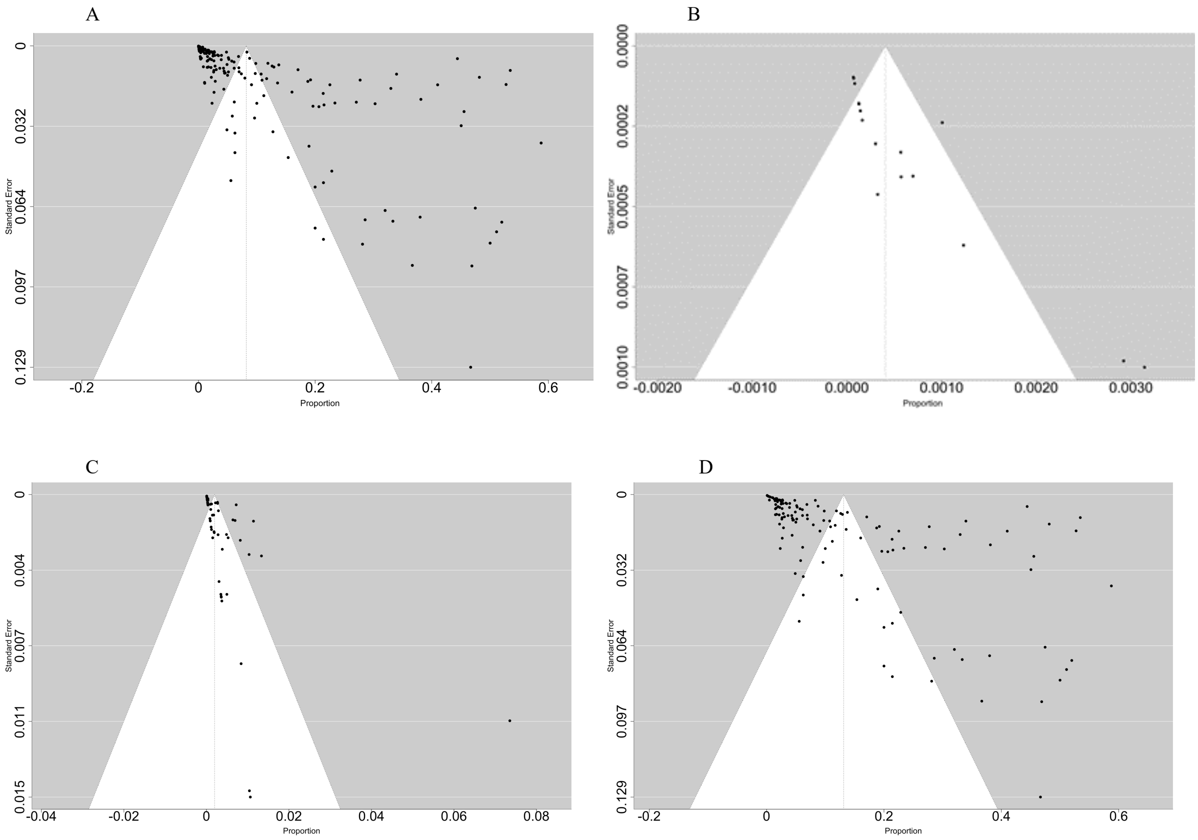

Figure S1: Funnel plots for standard error of the difference between microfilarial (mf) prevalence (number positive/examined) following the intervention period for: (A) the total number of records reporting parasitological information on this variable; (B) records reporting elimination of transmission; (C) records classified as close to elimination; (D) records reporting ongoing transmission. The black dots represent the mf prevalence (proportion) of individual studies. The unshaded area inside the diagonal dotted lines represents the region where 95% of the data points would lie if there were no systematic heterogeneity/publication bias. The dashed vertical line shows the average standardized mean difference of the microfilarial prevalence. The observed publication bias, indicated by studies deviating from the funnel plots to the shaded regions may be attributed to several reasons. One is the criteria employed for study inclusion, which selectively excluded foci characterized by treatment durations of less than ten years. Secondly, the exclusion of grey literature may have influenced the distinct shape of the funnel plot distribution. The heterogeneity evident in our studies, stemming from disparate population characteristics and variations in blackfly species, may contribute to the observed distribution patterns within the funnel plots.

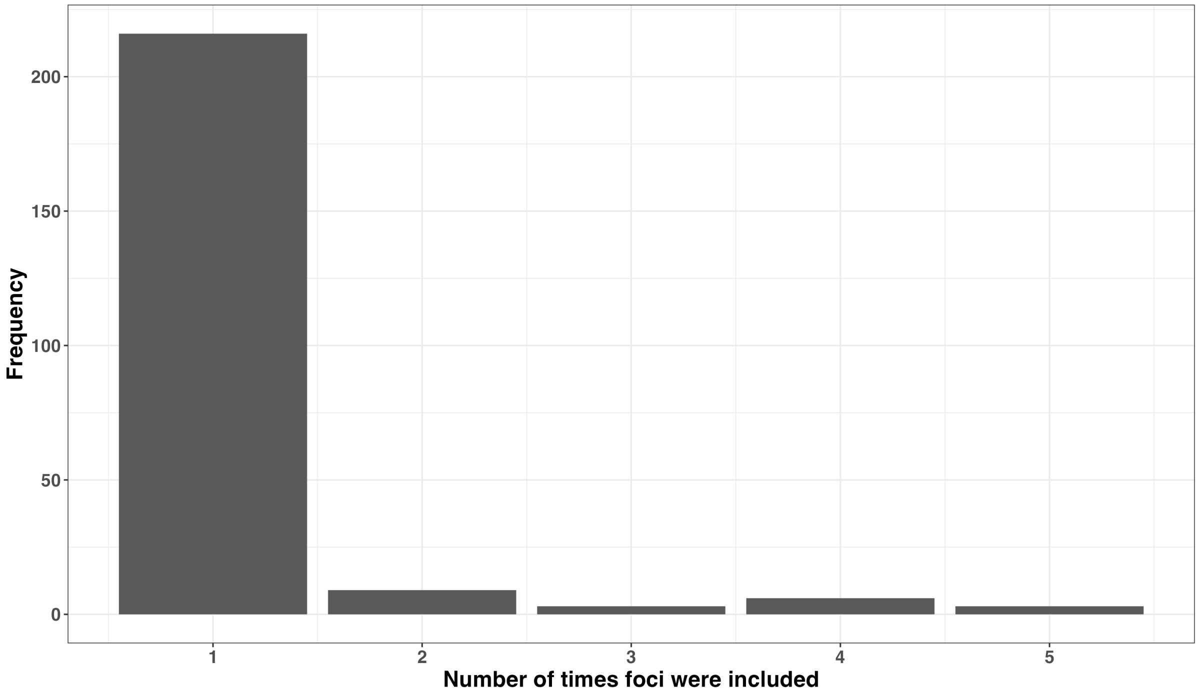

Figure S2: Frequency histogram showing the number of times the retrieved records from the 238 distinct endemic foci entered the analysis. Of the 282 records identified, 216 (77%) were included once; 10 (3.5%) were included two times (Abu Hamed, Asubende, Bandjoun, Kaduna, Kasese, Kebbi, Mahenge, Ogun, Plateau and Nasarawa); 3 (1%) were included three times (Bandja, River Gambia and Tukuyu); 6 (2%) four times (Bakoye, Bafang, Bafia, Bangangte, Foumbot and Kekem), and 3 (1%) five times (Bioko, Falémé and Massangam). Therefore, some records were classified into one or more transmission status categories, according to when epidemiological evaluations were conducted that reported ongoing transmission, close to elimination, or elimination of transmission.

Table S6: Characteristics of the 282 records (from 238 distinct onchocerciasis-endemic foci) that were included in the analysis

Author and year of publication	Year of study	Country	Subnational area / focus	Baseline endemicity	Years of MDA	Frequency MDA	Years biannual MDA	Therapeutic coverage of eligibles§	Years of therapeutic coverage ≥80% elig.	*Simulium spp.*	Vector control, no. yr	Trans-mission status	PTS
						West African region							
Borsboom et al., 2003[6]	2002	Burkina Faso	Dienkoa	Hyper-endemic	13	Annual	0	≥80%	–	*S. damno-sum* s.l.	Yes; 26 years	Ongoing transmission	–
Koudou et al., 2018[33]	2007–2016	Côte d'Ivoire	Oumé	Hyper-endemic	18	Annual, biannual in 1995–1996	1	<80%	–	*S. damno-sum* s.l.	Yes, 5 years	Close to elimination	–
Koudou et al., 2018[33]	2007–2016	Côte d'Ivoire	Yamoussoukro	Hypo-endemic	18	Annual	0	≥80%	–	*S. damno-sum* s.l.	Yes, 5 years	Close to elimination	–
Koudou et al., 2018[33]	2007–2016	Côte d'Ivoire	Odienné, Korhogo, Zuénoula	Meso-endemic	20	Annual, biannual in 1995–1996	1	≥80%	–	*S. damno-sum* s.l.	Yes, 5 years	Close to elimination	–
Koudou et al., 2018[33]	2007–2016	Côte d'Ivoire	Adzope, Boundiali, Mankono	Hyper-endemic	20	Annual, biannual in 1995–1996	1	<80%	–	*S. damno-sum* s.l.	Yes, at least 5 years	Close to elimination	–
Koudou et al., 2018[33]	2007–2016	Côte d'Ivoire	Agboville, Tengrela, Issia	Hyper-endemic	20	Annual, biannual in 1995-1996	1	–	–	*S. damno-sum* s.l.	Yes, 5 years	Close to elimination	–
Koudou et al., 2018[33]	2007–2016	Côte d'Ivoire	Tiassalé, Bouaké	–	16,18	Annual, biannual in 1995–1996	1	<80%	–	*S. damno-sum* s.l.	Yes, 5 years	Ongoing transmission	–
Koudou et al., 2018[33]	2007–2016	Côte d'Ivoire	Daloa, Man, Danané, Seguela, Bondoukou, Bouna, Bèoumi, Bouaflé, Biankouma, Alépé	Meso-endemic	16-18	Annual, biannual in 1995–1996	1	≥80%	–	*S. damno-sum* s.l.	Yes, 5 years	Ongoing transmission	–
Koudou et al., 2018[33]	2007–2016	Côte d'Ivoire	Touba, Agnibilékro, Abengourou, Dabakala, Dimbokro, Daoukro, M'Bahiakro,	Hyper-endemic	20	Annual, biannual in 1995–1996	1	≥80%	–	*S. damno-sum* s.l.	Yes, 5 years	Ongoing transmission	–

			Katiola, Bocanda										
Koudou et al., 2018[33]	2007–2016	Côte d'Ivoire	Soubré, Sassandra, Ferkéssédougo Ouangolo	Hyper-endemic	20	Annual, biannual in 1995–1996	1	–	–	*S. damno-sum* s.l.	Yes, 5 years	Ongoing transmission	–
Borsboom et al., 2003[6]	2002	Ghana	Asubende	Hyper-endemic	14	Annual	0	<80%	–	*S. damno-sum* s.l.	Yes; 11 years	Ongoing transmission	–
Borsboom et al., 2003[6]	2002	Ghana	Bui Gorge	Hyper-endemic	14	Annual, quarterly frequency in 1994–1996	2 quarterly	≥80%	NA	*S. damno-sum* s.l.	Yes; 21 years	Close to elimination	–
Osei-Atweneboana et al., 2007[43]	2004–2005	Ghana	Kintampo	–	17	Annual	0	<80%	–	*S. damno-sum* s.l.	No	Ongoing transmission	–
Osei-Atweneboana et al., 2007[43]	2004–2005	Ghana	Atebubu	–	17	Annual	0	<80%	–	*S. damno-sum* s.l.	No	Close to elimination	–
Osei-Atweneboana et al., 2007[43]	2004–2005	Ghana	Nkoranza	–	12	Annual	0	<80%	–	*S. damno-sum* s.l.	No	Close to elimination	–
Osei-Atweneboana et al., 2007[43]	2004–2005	Ghana	East Gonja	–	11	Annual	0	<80%	–	*S. damno-sum* s.l.	No	Close to elimination	–
Lamberton et al., 2015[38]	2009–2011	Ghana	Asukawkaw Ferry, Dodi Papase	Hyper-endemic	18	Annual	0	<80%	–	*S. damno-sum* s.l.	Yes, 5 and 15 years respecti vely	Ongoing transmission	–
Lamberton et al., 2015[38]	2009–2011	Ghana	Pillar 83/Djodji	Hyper-endemic	18	Annual, biannual in 1993-1997	4	≥80%	–	*S. damno-sum* s.l.	Yes, 15 years	Ongoing transmission	–
Lamberton et al., 2015[38]	2009–2011	Ghana	Asubende	Hyper-endemic	14	Biannual	2	≥80%	–	*S. damno-sum* s.l.	Yes, 13 years	Ongoing transmission	–
Lamberton et al., 2015[38]	2009–2011	Ghana	Agborlekame	Hyper-endemic	24	Biannual	2	<80%		*S. damno-sum* s.l.	Yes, 27 years	Ongoing transmission	–
Garms et al., 2015[12]	2006, 2013–2014	Ghana	Toll bridge, Buabenso, Adwuman, Akropong, Kyekyewere, Amoafo,	Hyper-endemic	10–14	Annual	0	–	–	*S. damno-sum* s.l.<br><br>*S. sancti-pauli* affected	No	Ongoing transmission	–

			Badowa, Foso, Nyaduom, Asaman, Awisam, Asma camp, Zion camp, Buabin & Imbraim							by gold-mining in river Pra			
Otabil et al., 2019[46]	2017	Ghana	Nkoranza North	–	19	Annual	0	_	–	*S. damno-sum* s.l.	No	Ongoing transmission	–
Otabil et al., 2023[66]	2020–2021	Ghana	Tain District, Wenchi Municipality	Meso-endemic	27	Biannual	12 (in Tain), 3 (in Wenchi)	≥80%	–	*S. damno-sum* s.l.	Yes; 20 years in Tain, 4 years in Wenchi	Ongoing transmission	–
Biritwum et al., 2021[5]	2016	Ghana	Ashanti, Brong Ahafo, Central, Eastern, Greater Accra, Northern, Upper East, Upper West, Volta, Western	Hyper-endemic	24	Biannual since 2009 in villages along some river basins according to risk	7	<80%<br>≥80%	 3	*S. damno-sum* s.l.	Yes; 28 years	Close to elimination	–
Borsboom et al., 2003[6]	2002	Guinea	Milo & Sankarani	Hyper-endemic	12	Annual	0	<80%	–	*S. damno-sum* s.l.	Yes; 12 years	Ongoing transmission	–
Borsboom et al., 2003[6]	2002	Guinea & Mali	Bafing	Meso-endemic	12	Annual	0	<80%	–	*S. damno-sum* s.l.	No	Ongoing transmission	–
Borsboom et al., 2003[6]	2002	Guinea & Mali	River Bakoye	Hyper-endemic	12	Annual	0	<80%	–	*S. damno-sum* s.l.	No	Ongoing transmission	–
Borsboom et al., 2003[6]	2002	Guinea & Senegal	River Mako & River Senegal	Hyper-endemic	12	Biannual since 1989	12	≥80%	–	*S. damno-sum* s.l.	No	Ongoing transmission	–
Borsboom et al., 2003[6]	2002	Mali	Tienfala	Hyper-endemic	14	Annual	0	≥80%	–	*S. damno-sum* s.l.	Yes- 7 years	Ongoing transmission	–
Borsboom et al., 2003[6]	2002	Mali & Senegal	River Falémé	Hyper-endemic	12	Annual	–	≥80%	–	*S. damno-sum* s.l.	No	Ongoing transmission	–
Diawara et al., 2009[7]	2006–2008	Mali	River Bakoye	Hypo- (23%) to hyper-endemic (38%)[64]	15	Annual	0	<80% (38–78%)<br>≥80% (73–83%)	 8	*S. damno-sum* s.l.	No	Elimination of transmission	Yes, in 2009–2011 by Traore et al, 2012[30]

Diawara et al., 2009[7]	2006–2008	Mali & Senegal	River Falémé	Hypo- to hyper-endemic	16	Annual	0	<80% (38–77%)<br>≥80% (77–81%)	 8	*S. damno-sum* s.l.	No	Elimination of transmission	Yes, in 2009–2011 by Traore et al, 2012[30]
Diawara et al., 2009[7]	2006–2008	Senegal	River Gambia	Hypo- (7%) to hyper-endemic (50%)[64]	17	Biannual since 1989	17	<80%<br>≥80%	 8	*S. damno-sum* s.l.	No	Elimination of transmission	Yes, in 2009–2011 by Traore et al, 2012[30]
Dolo et al., 2021[9]	2017–2018	Mali	River Bakoye	Hyper-endemic	24–25	Annual	0	–	–	*S. damno-sum* s.l.	Yes	Elimination of transmission	LF co-endemicity, PTS not conducted
Dolo et al., 2021[9]	2017–2018	Mali	River Falémé	Meso-endemic	24–25	Annual	0	–	–	*S. damno-sum* s.l.	Yes	Elimination of transmission	LF co-endemicity, PTS not conducted
Anosike et al., 2007[2]	2005	Nigeria	Abia	Meso-endemic	11	Annual	0	–	–	*S. damno-sum* s.l.	No	Ongoing transmission	–
Anosike et al., 2007[2]	2005	Nigeria	Imo	Meso-endemic	12	Annual	0	–	–	*S. damno-sum* s.l.	No	Ongoing transmission	–
Anosike et al., 2007[2]	2005	Nigeria	Enugu	Meso-endemic	10	Annual	0	–	–	*S. damno-sum* s.l.	No	Ongoing transmission	–
Anosike et al., 2007[2]	2005	Nigeria	Anambra	Meso-endemic	10	Annual	0	–	–	*S. damno-sum* s.l.	No	Ongoing transmission	–
Kogi and Bulus, 2008[31]	2002	Nigeria	Galadimawa	Hyper-endemic	12	Annual	0	–	–	*S. damno-sum* s.l.	No	Ongoing transmission	–
Okon and Ilugbiyin, 2008[42]	2006	Nigeria	Cross-River State, Aninegeye	Hyper-endemic	14	Annual	0	–	–	*S. damno-sum* s.l.	No	Ongoing transmission	–
Sam-Wobo et al., 2012[49]	2009	Nigeria	Oyo & Ogun States, Ogun river system	–	14-15	Annual	0	–	–	*S. damno-sum* s.l.	No	Ongoing transmission	–
Tekle et al., 2012[56]	2008	Nigeria	Kaduna State, Birnin Gwari	Hypo- to hyper-endemic (Median = meso-endemic)	15	Annual	0	≥80% Median = 77.7 (74.4–80.4)%	15–17	*S. damno-sum* s.l.	No	Close to elimination	–
Tekle et al., 2012[56]	2008	Nigeria	Kaduna State, Kauru	Hypo- to hyper-endemic	15	Annual	0	≥80%	15–17	*S. damno-sum* s.l.	No	Close to elimination	–

				(Median = meso-endemic)				Median = 77.7 (74.4–80.4)%					
Tekle et al., 2012[56]	2008	Nigeria	Kaduna State, Lere	Hypo- to hyper-endemic (Median = meso-endemic)	15	Annual	0	≥80% Median = 77.7 (74.4–80.4)%	15–17	*S. damnosum* s.l.	No	Close to elimination	–
Osue et al., 2013[44]	2011	Nigeria	Kaduna State, Kachia Local Government Area	Meso-endemic	18	Annual	0	≥80%	–	*S. damnosum* s.l.	No	Close to elimination	–
Evans et al., 2014[10]	2009	Nigeria	Nasarawa & Plateau States	Meso-endemic	17	Annual	0	≥80%	14	*S. damnosum* s.l.	No	Close to elimination	–
Sufi and Zainab, 2015[53]	2012	Nigeria	Kano State, Zainabi & Ririwai	–	10	Annual	0	–	–	*S. damnosum* s.l.	No	Ongoing transmission	–
Tekle et al., 2016[57]	2013	Liberia	Lofa, Bong, Nimba	Meso-endemic	14	Annual	0	<80%	–	*S. damnosum* s.l.	No	Ongoing transmission	–
Tekle et al., 2016[57]	2012, 2013	Nigeria	Adamawa, FCT, Kano, Kebbi, Niger, Osun, Oyo, Plateau Nasarawa	Meso- to hyper-endemic	10–14	Annual	0	<80%	–	*S. damnosum* s.l.	No	Close to elimination	–
Tekle et al., 2016[57]	2009, 2012, 2013	Nigeria	Cross River, Ebonyi, Enugu Anambra, Ekiti, Kwara &Tabara	Meso- to hyper-endemic	10–14	Annual	0	<80%	–	*S. damnosum* s.l.	No	Ongoing elimination	–
Tekle et al., 2016[57]	2012	Nigeria	Kaduna State	Hyper-endemic	21	Annual	0	<80%	–	*S. damnosum* s.l.	No	Close to elimination	–
Osue Hudu, 2017[45]	2004	Nigeria	Gurara River basin	–	10	Annual	0	<80%	–	*S. damnosum* s.l.	No	Ongoing transmission	–
Surakat et al., 2018[54]	2015	Nigeria	Ogun State: Okeda, Abeokuta North and South, Imeko-Afon, Ewekoro, Ifo, Obafemi-Owode, Yewa North	Meso-endemic	10	Annual	0	<80%	–	*S. damnosum* s.l.	No	Ongoing transmission	–

Siewe Fodjo et al., 2019[52]	2018	Nigeria	Imo River basin	–	13	Biannual	6	≥80%	–	*S. damno-sum* s.l.	No	Ongoing transmission	–
Richards et al., 2020[48]	2017	Nigeria	Nasarawa & Plateau States	Meso- to hyper-endemic	16,19	Annual	0	≥80%	22	*S. damno-sum* s.l.	No	Elimination of transmission	Yes, in 2019-2021 by Miri et al., 2023[71]
Chikezie et al., 2023[67]	2018–2019	Nigeria	Cross River State: Agbokim, Aningeje, Ekong Anaku, Orimekpang	–	27	Annual	–	<80%	–	*S. damno-sum* s.l.	No	Close to elimination (3/4) to Ongoing transmission (1/4 villages)	–
Ekpo et al., 2022[68]	2020–2021	Nigeria	Enugu & Ogun States: Amokwe, Eziobodo, Obinagu/ Eziama, Ugwuorie, Umuezemanna, Umunnakwe, Abule Aje, Abule Peter, Adeaga, Ibara Afon, Ibaro, Idode, Imomo, Isara, Olokemeji, Olowo	Hypo- to hyper-endemic	11–26	Annual	–	≥80%	–	*S. damno-sum* s.l.	No	Close to elimination (1/16) to Ongoing transmission (15/16 villages)	–
Anagbogu et al., 2022[72]	2017	Nigeria	Cross River, Taraba & Yobe States: Bade, Ikom, Bekwara, Gashaka, Karim-Lamido	Hypo- to hyper-endemic	–	Annual	–	≥80%	–	*S. damno-sum* s.l.	No	Close to elimination	–
Isiyaku et al., 2022[75]	2016 (human) 2017–2020 (vector)	Nigeria	Kaduna, Kebbi, Zamfara States	Hypo- to hyper-endemic	20–28	Annual	–	≥80%	12–20	*S. damno-sum* s.l.	No	Elimination of transmission	–

Wilson et al., 2016[61]	2014	Senegal	River Falémé & River Kaolakabe	–	16	Annual	0	<80%	–	*S. damno-sum* s.l.	No	Close to elimination	–
Wilson et al., 2016[61]	2014	Senegal	River Gambia	Hyper-endemic	18	Biannual since 1990	24	<80%	–	*S. damno-sum* s.l.	No	Close to elimination	–
Borsboom et al., 2003[6]	2002	Togo	Titira & Kouporgou	Hyper-endemic	13	Annual	0	≥80%	–	*S. damno-sum* s.l.	Yes, 24 years	Ongoing transmission	–
Komlan et al., 2018[32]	2015	Togo	Central Togo	Hyper-endemic	25	Annual, with biannual from 2003–2012 in SIZ	9	≥80%	12	*S. damno-sum* s.l.	Yes, 26 years	Ongoing transmission	–
Komlan et al., 2018[32]	2015	Togo	Northern Togo	Hyper-endemic	25	Annual, with biannual from 2003–2012 in SIZ	9	≥80%	12	*S. damno-sum* s.l.	Yes, 15 years	Ongoing transmission	–
Johanns et al., 2022[18]	2019	Togo	Region Centrale	Hyper-endemic	30	Annual, biannual from 2003-2012 in SIZ	9	–	–	*S. damno-sum* s.l.	Yes, 31 years	Ongoing transmission	–
Central African region													
Seidenfaden et al., 2001[50]	1996–1998	Cameroon	Vina du Nord	Hyper-endemic	10	Annual	0	<80%	–	*S. damno-sum* s.l.	No	Ongoing transmission	–
Borsboom et al., 2003[6]	2002	Cameroon	Vina Valley	Hyper-endemic	13	Annual	0	<80%	–	*S. damno-sum* s.l.	No	Ongoing transmission	–
Katabarwa et al., 2008[22]	2005	Cameroon	Bangangte, Foumbot, Bafang, Kekem, Bandja	Hyper-endemic	10	Annual	0	≥80%	–	*S. damno-sum* s.l.	No	Ongoing transmission	–
Katabarwa et al., 2008[22]	2005	Cameroon	Kekem	Hyper-endemic	10	Annual	0	<80%	–	*S. damno-sum* s.l.	No	Ongoing transmission	–
Katabarwa et al., 2011[23]	2008-2009	Cameroon	Rey-Bouba Health District	Hyper-endemic	12	Annual	0	≥80%	6	*S. damno-sum* s.l.	No	Ongoing transmission	–
Katabarwa et al., 2011[23]	2008-2010	Cameroon	Tcholliré	Meso-endemic	12	Annual	0	≥80%	7	*S. damno-sum* s.l.	No	Close to elimination	–
Katabarwa et al., 2011[23]	2008-2009	Cameroon	Touboro Health District	Hyper-endemic	17	Annual	0	≥80%	6	*S. damno-sum* s.l.	No	Ongoing transmission	–
Katabarwa et al., 2013a[25]	2011	Cameroon	Bafang, Baham, Bandja, Bangangte, Foumbot, Kekem, Penka-Michel,	Hyper-endemic	14	Annual	0	≥80%	8	*S. damno-sum* s.l.	No	Ongoing transmission	–

			Foumban, Kouoptamo, Malantouen, Massangam										
Katabarwa et al., 2013a[25]	2011	Cameroon	Foumban, Folap village	–	14	Annual	0	≥80%	8	*S. damno-sum* s.l.	No	Close to elimination	–
Wanji et al., 2015[60]	2011-2012	Cameroon	Manyu River, Mungo & Meme basin	Hyper-endemic	10–14	Annual	0	<80%	–	*S. damno-sum* s.l.	No	Ongoing transmission	–
Tekle et al., 2016[57]	2010-2012	Cameroon	North Tcholliré, Centre 1, Littoral 2, Western Province, Adamawa II, South West 1, South West 2	Hyper-endemic	11	Annual	0	<80%	–	*S. damno-sum* s.l.	No	Ongoing transmission	–
Tekle et al., 2016[57]	2010	Cameroon	North Toubouro	–	21	Annual	0	<80%	–	*S. damno-sum* s.l.	No	Ongoing transmission	–
Kamga et al., 2016[19]	2015	Cameroon	Bafia & Yabassi Health Districts	Hyper-endemic	15,16	Annual	0	<80%	–	*S. damno-sum* s.l.	No	Ongoing transmission	–
Kamga et al., 2017[20]	2015	Cameroon	Bafang, Bandja, Bandjoun, Bangangte, Foumbot, Kekem, Massangam Health Districts	Hyper-endemic	19	Annual	0	<80% except Bandja	–	*S. damno-sum* s.l.	No	Ongoing transmission	–
Kamga et al., 2018[21]	2015	Cameroon	Bafia, Foumbot-Massangam, Yabassi Health Districts	Hyper-endemic	15	Annual	0	≥80% except Yabassi	3	*S. damno-sum* s.l.	No	Ongoing transmission	–
Bakajika et al., 2018[4]	2015-2016	Cameroon	Massangam	Hyper-endemic	20	Annual	0	≥80%	–	*S. damno-sum* s.l.	No	Ongoing transmission	–
Abong et al., 2020[1]	2016–2017	Cameroon	Bafia Health District	–	>20	Annual	0	≥80%	–	*S. damno-sum* s.l.	No	Ongoing transmission	–
Abong et al., 2020[1]	2016–2017	Cameroon	Melong	Hyper-endemic	10	Annual	0	≥80%	–	*S. damno-sum* s.l.	No	Ongoing transmission	–
Aza'ah et al., 2020[3]	2018	Cameroon	Ndikinimeki	Hyper-endemic	20	Annual	0	<80%	–	*S. damno-sum* s.l.	No	Ongoing transmission	–
Nyagang et al., 2020[41]	2017-2018	Cameroon	Tombel	Hyper-endemic	15	Annual	0	≥80%	13	*S. damno-sum* s.l.	No	Ongoing transmission	–

Shintouo et al., 2020[51]	2019	Cameroon	Bandjoun, Bangangte & Foumbot	–	23	Annual	0	–	–	*S. damnosum* s.l.	No	Close to elimination	–
Shintouo et al., 2020[51]	2019	Cameroon	Bafang Kekem & Massangam	–	23	Annual	0	–	–	*S. damnosum* s.l.	No	Ongoing transmission	–
Forrer et al., 2021[11]	2017	Cameroon	Meme River basin	–	>15	Annual	0	<80%	–	*S. damnosum* s.l.	No	Ongoing transmission	–
Djune-Yemeli et al., 2022[19]	2019	Cameroon	Bafia Health district	Meso-endemic	20	Annual	0	–	–	*S. damnosum* s.l.	No	Ongoing transmission	–
Atekem et al., 2022[65]	2019	Cameroon	Massangam	Hypo-endemic	23	Biannual	5	<80%	–	*S. damnosum* s.l.	Yes, 2 years	Ongoing transmission	–
Efon-Ekanguouo et al., 2023[70]	2021	Cameroon	Babeta, Biatsota, Ngongol, Nyamanga 1, Boneck, Kiboum 1, Kiboum 2		>20	Annual	–	≥80%	–	–	No	Ongoing transmission	–
Ekanya et al., 2022[73]	2017	Cameroon	Berenge, Betenge, Big Massaka, Bombele, Kwakwa, Muramba 1, Small Massaka	Hyper- to holo-endemic		Annual	–	≥80%	–	*S. damnosum* s.l.	No	Ongoing transmission	–
Domche et al., 2022[74]	2019-2020	Cameroon	Bayomen, Biatsota	Hyper- to holo-endemic	24	Annual	–	≥80%	–	–	No	Ongoing transmission	–
Tekle et al., 2016[57]	2010	Central African Republic	Basse-Kotto	Hyper-endemic	11	Annual	0	<80%	–	*S. damnosum* s.l.	No	Ongoing transmission	–
Tekle et al., 2016[57]	2012	Central African Republic	Ouaham Pende & Ouaka	Hyper-endemic	13	Annual	0	<80%	–	*S. damnosum* s.l.	No	Close to elimination	–
Yaya et al., 2014[62]	2010	Central African Republic	Gami	Hyper-endemic	22	Annual	0	<80%	–	*S. damnosum* s.l.	No	Ongoing transmission	–
Tekle et al., 2016[57]	2012-2014	Chad	Logon Occidental, Logon Oriental, Mandoul, Mayo Kebbi East,	Hyper-endemic	16	Annual	0	<80%	–	*S. damnosum* s.l.	No	Close to elimination	–

			Moyen Chari, Tandjile										
Tekle et al., 2016[57]	2011	Congo	Bouenza	Meso-endemic	10	Annual	0	<80%	–	*S. damnosum* s.l.	No	Close to elimination	–
Tekle et al., 2016[57]	2012	Democratic Republic of Congo	Uele	Hyper-endemic	10	Annual	0	<80%	–	*S. damnosum* s.l.	No	Ongoing transmission	–
Traoré et al., 2009[58]	2007–2008	Equatorial Guinea	Bioko Island	Hyper-endemic	10	Annual	0	<80%	–	Bioko form of *S. yahense* (*S. damnosum* s.l.)	Yes, 2003, 2005, vector eliminated	Ongoing transmission	–
Hernández-González et al., 2016[15]	2014	Equatorial Guinea	Bioko Island	Hyper-endemic	19	Annual	0	<80%	–	As above	As above	Close to elimination	–
Moya et al., 2016[39]	2014	Equatorial Guinea	Bioko Island	Hyper-endemic	25	Annual	0	–	–	As above	As above	Close to elimination	–
Ta et al., 2018[55]	2014	Equatorial Guinea	Bioko Island	Hyper-endemic	16	Annual	0	–	–	As above	As above	Close to elimination	–
Herrador et al., 2018[16]	2016-2017	Equatorial Guinea	Bioko Island	Hyper-endemic	26	Annual	0	–	–	As above	As above	Elimination of transmission	Not conducted; recommended
Eastern African region													
Tekle et al., 2016[57]	2013	Ethiopia	North Gondar	Hyper-endemic	10	Annual	0	<80%	–	*S. damnosum* s.l.	No	Close to elimination	–
Tekle et al., 2016[57]	2011	Ethiopia	Kafa, Shekka & Bench Maji	Hyper-endemic	10	Annual	0	<80%	–	*S. damnosum* s.l.	No	Ongoing transmission	–
Gebrezgabiher et al., 2020[13]	2017-2018	Ethiopia	Yeki	Hyper-endemic	15	Biannual	7	≥80%	6	–	No	Close to elimination	–
Katabarwa et al., 2020a[29]	2014-2017	Ethiopia	Metema	Hyper-endemic	14	Annual, biannual in 2016	1	≥80%	14	*S. damnosum* s.l.	No	Elimination of transmission	
Tekle et al., 2016[57]	2012	Malawi	Malawi extension & Thyolo Muanza	Meso-endemic	12<br>13	Annual	0	<80%	–	*S. damnosum* s.l.	No	Close to elimination	–
Higazi et al., 2013 [17]	2011	Sudan	Abu Hamed	Meso-endemic	13	Biannual	5	≥80%	2	*S. damnosum* s.l.	No	Elimination of transmission	Yes, in 2014–2015. Zarroug et al., 2016[39]

Katabarwa et al., 2020a [29]	2014–2017	Sudan	Galabat	Hyper-endemic	10	Annual, biannual from 2011–2014	3	≥80%	14	*S. damnosum* s.l.	No	Elimination of transmission	–
Mweya et al., 2007[40]	2004	Tanzania	Tukuyu	Hyper-endemic	10	Annual	0	–	–	*S. damnosum* s.l.	No	Close to elimination	–
Tekle et al., 2016[57]	2012	Tanzania	Tanga & Tukuyu	Meso- to hyper-endemic	11<br>12	Annual	0	<80%	–	*S. damnosum* s.l.	No	Close to elimination	–
Tekle et al., 2016[57]	2009, 2011, 2012	Tanzania	Kilosa, Mahenge & Ruvuma	Meso-endemic	10–12	Annual	0	<80%	–	*S. damnosum* s.l.	No	Ongoing transmission	–
Tekle et al., 2016[57]	2013	Tanzania	Tunduru	Hyper-endemic	18	Annual	0	<80%	–	*S. damnosum* s.l.	No	Close to elimination	–
Paulin et al., 2017[47]	2015	Tanzania	Tukuyu	Hypo- to hyper-endemic	15	Annual	0	<80%	–	*S. damnosum* s.l.	Yes, 3 years	Ongoing transmission	–
Hendy et al., 2018[14]	2015–2016	Tanzania	Mahenge	Hyper-endemic	19	Annual	0	<80%	–	*Simulium neavei*	No	Ongoing transmission	–
Mshana et al., 2023[64]	2022	Tanzania	Ulanga (Morogoro)	Hyper-endemic	20	Biannual	3	≥80%	–	*S. damnosum* s.l.	No	Ongoing transmission	–
Bhwana et al., 2023[69]	2021	Tanzania	Mahenge: Mdindo, Msogezi, Mzelezi, Sali	Hyper- to holo-endemic	24	Biannual	2	<80%<br>≥80%	1	*S. damnosum* s.l., *S. neavei*	No	Ongoing transmission	–
Katabarwa et al., 2008[22]	2005	Uganda	Kasese, Kisoro, Mbale, Moyo, Nebbi	Hyper-endemic	13	Annual	0	≥80%	–	–	No	Ongoing transmission	–
Katabarwa et al., 2012[24]	2010	Uganda	Wadelai	Meso-endemic	18	Annual from 1993–2005, biannual from 2006–2010[65]	5	≥80%	15	*Simulium neavei*	No, disappeared	Elimination of transmission	Not conducted; later reported for 2017–2020[65]
Katabarwa et al., 2013b[26]	2010–2011	Uganda	Nyagak-Bondo	Hyper-endemic	15–19	Annual from 1993–2011, biannual from 2012[65]	7	≥80%	12	*Simulium neavei*	No	Ongoing transmission	–
Katabarwa et al., 2014[27]	2007–2011	Uganda	Mount Elgon	Hyper-endemic	18	Annual from 1994–2006, biannual	5	≥80%	4	*Simulium neavei*	Yes, 1.25 years	Elimination of transmission	Not conducted; later reported for

						from 2007–2011[65]							2012–2015[65]
Katabarwa et al., 2016[28]	2012–2015	Uganda	Imaramagambo	Hypo- to meso-endemic	22	Annual from 1991–2012[65]	0	<80%	–	*Simulium neavei*	No, disap-peared	Elimination of transmission	Yes; reported for 2013–2016[65]
Lakwo et al., 2013[34]	2010	Uganda	Itwara	Hyper-endemic	21	Annual from 1991–2011[65]	0	≥80%	17	*Simulium neavei*	Yes, 1 year, elimina-tion	Elimination of transmission	Not conducted; recommen-ded; later reported for 2012–2015[65]
Lakwo et al., 2015[35]	2012	Uganda	Mpamba-Nkusi	Holo-endemic	18	Annual from 1995–2008, biannual from 2009–2012[65]	4	≥80%	18	*Simulium neavei*	Yes, 2–7 years, elimina-tion	Elimination of transmission	Not conducted; recommen-ded; later reported for 2013–2016[65]
Lakwo et al., 2017[36]	2013	Uganda	Kashoya-Kitomi	Hyper-endemic	23	Annual from 1991–2006, biannual from 2007–2013[65]	7		12	*Simulium neavei*	Yes, 3–5 years, elimina-tion	Elimination of transmission	Not conducted; recommen-ded; later reported for 2014–2017[65]
Lakwo Luroni et al., 2017[37]	2014	Uganda	Obongi	Hyper-endemic	21	Annual from 1993–2014[65]	0	≥80%	12	*S. damno-sum* s.l.	No, disap-peared	Elimination of transmission	Not conducted; recommen-ded with LF; later reported for 2016–2019[65]
Katabarwa et al., 2020b[30]	2014–2017	Uganda	Wambabya-Rwamarongo	–	23	Annual from 1991–2006, biannual from 2007–2013[65]	7	≥80%	9	*Simulium neavei*	Yes, 3 years, elimina-tion	Elimination of transmission	Yes; reported for 2014–2017[65]
Tekle et al., 2016[57]	2012	Uganda	Kasese	Hyper-endemic	14	Annual	0	<80%	–	*S. damno-sum* s.l.	No	Close to elimination	–

Tekle et al., 2016[57]	2009 2012	Uganda	Adjumani Mojo & Arua Nebbie	Hyper-endemic	10 13	Annual	0	<80%	–	*S. damnosum* s.l.	No	Ongoing transmission	–

§ For papers that had reported coverage of total population, this value was converted into coverage of eligible population by multiplying it by 1.23 (80/65), as treating 80% of the eligible population is approximately equivalent to treating 65% of the total population.
PTS: Post-treatment surveillance; SIZ: Special Intervention Zone; s.l.: sensu lato.

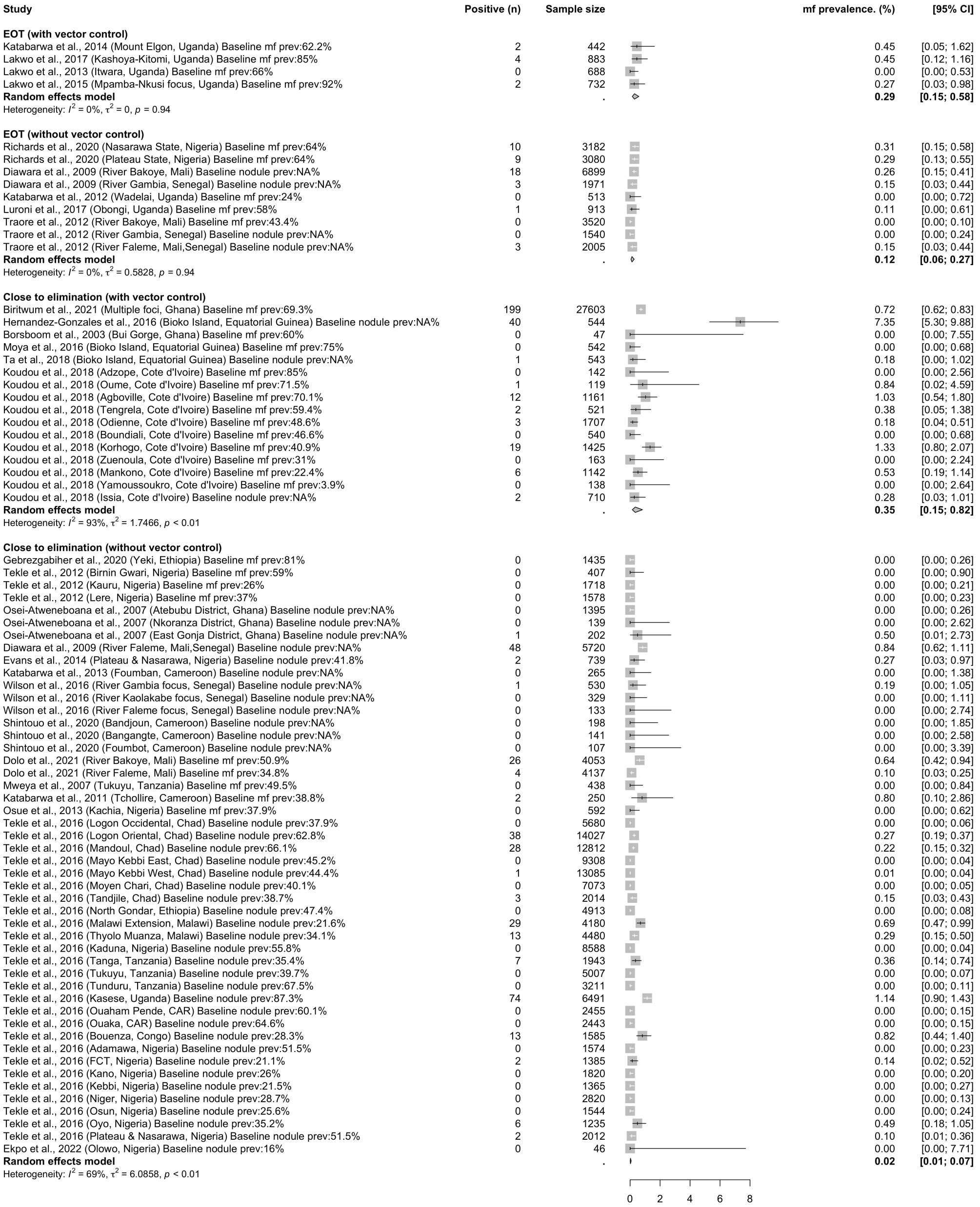

Figure S3: **Forest plots of microfilarial (mf) prevalence reported by studies conducting parasitological evaluations to measure the impact of interventions for records classified as reporting elimination of transmission (EOT) or being close to EOT with or without vector control.** Records reporting EOT had achieved proposed serological/entomological thresholds,[11,19] and those classified as close to elimination had mf prevalence <1% at the time of the evaluations, but had not conducted sero- or xenomonitoring, or had not met proposed thresholds. Some records appear in both groups (e.g. Plateau and Nasarawa in Nigeria) depending on the time the evaluations took place. The references used are described in page 10. The grey boxes denote the effect sizes of studies and the horizontal lines are the 95%CIs.

Study	Positive (n)	Sample size	mf prevalence. (%)	[95% CI]
**Ongoing transmission (with vector control)**				
Komlan et al., 2018 (Central Togo, Togo) Baseline nodule prev:NA%	83	1455	5.70	[4.57; 7.02]
Komlan et al., 2018 (Northern Togo, Togo) Baseline nodule prev:NA%	83	1455	5.70	[4.57; 7.02]
Borsboom et al., 2003 (Tienfala, Mali) Baseline mf prev:80%	1	18	5.56	[0.14; 27.29]
Borsboom et al., 2003 (Titira & Kouporgou, Togo) Baseline mf prev:89%	28	59	47.46	[34.30; 60.88]
Borsboom et al., 2003 (Milo & Sankarani, Guinea) Baseline mf prev:87%	17	170	10.00	[5.93; 15.53]
Johanns et al., 2022 (Region Centrale, Togo) Baseline mf prev:52.2%	2	124	1.61	[0.20; 5.70]
Koudou et al., 2018 (Touba, Cote d'Ivoire) Baseline mf prev:66.7%	89	1709	5.21	[4.20; 6.37]
Koudou et al., 2018 (Man, Cote d'Ivoire) Baseline mf prev:65.9%	265	1553	17.06	[15.22; 19.03]
Koudou et al., 2018 (Alepe, Cote d'Ivoire) Baseline mf prev:64%	163	358	45.53	[40.29; 50.85]
Koudou et al., 2018 (Soubre, Cote d'Ivoire) Baseline mf prev:62.5%	119	393	30.28	[25.77; 35.09]
	16	358	4.47	[2.58; 7.16]
Koudou et al., 2018 (Sassandra, Cote d'Ivoire) Baseline mf prev:57%	99	463	21.38	[17.73; 25.40]
Koudou et al., 2018 (Danane, Cote d'Ivoire) Baseline mf prev:53.6%	94	160	58.75	[50.71; 66.46]
Koudou et al., 2018 (Bouake, Cote d'Ivoire) Baseline mf prev:51.3%	8	487	1.64	[0.71; 3.21]
Koudou et al., 2018 (Daloa, Cote d'Ivoire) Baseline mf prev:50.9%	4	139	2.88	[0.79; 7.20]
Koudou et al., 2018 (Tiassale, Cote d'Ivoire) Baseline mf prev:43.4%	22	513	4.29	[2.71; 6.42]
	107	3914	2.73	[2.25; 3.29]
Koudou et al., 2018 (Abengourou, Cote d'Ivoire) Baseline mf prev:42.6%	81	754	10.74	[8.62; 13.17]
Koudou et al., 2018 (Seguela, Cote d'Ivoire) Baseline mf prev:40.9%	23	448	5.13	[3.28; 7.60]
Koudou et al., 2018 (Dabakala, Cote d'Ivoire) Baseline mf prev:38.6%	12	422	2.84	[1.48; 4.91]
Koudou et al., 2018 (Dimbokro, Cote d'Ivoire) Baseline mf prev:33%	7	265	2.64	[1.07; 5.37]
Koudou et al., 2018 (Bondoukou, Cote d'Ivoire) Baseline mf prev:31.5%	56	514	10.89	[8.34; 13.91]
Koudou et al., 2018 (Bouna, Cote d'Ivoire) Baseline mf prev:30.5%	52	1859	2.80	[2.10; 3.65]
Koudou et al., 2018 (Daoukro, Cote d'Ivoire) Baseline mf prev:30.2%	28	250	11.20	[7.57; 15.78]
Koudou et al., 2018 (M'Bahiakro, Cote d'Ivoire) Baseline mf prev:29.8%	203	1570	12.93	[11.31; 14.69]
	6	138	4.35	[1.61; 9.22]
	16	719	2.23	[1.28; 3.59]
Koudou et al., 2018 (Ouangolo, Cote d'Ivoire) Baseline mf prev:26.2%	75	2188	3.43	[2.71; 4.28]
Koudou et al., 2018 (Katiola, Cote d'Ivoire) Baseline mf prev:13.3%	65	2623	2.48	[1.92; 3.15]
Koudou et al., 2018 (Bocanda, Cote d'Ivoire) Baseline mf prev:5.3%	10	104	9.62	[4.71; 16.97]
Koudou et al., 2018 (Biankouma, Cote d'Ivoire) Baseline nodule prev:NA%	57	276	20.65	[16.03; 25.91]
Atekem et al., 2022 (Massangam, Cameroon) Baseline mf prev:35.7%	108	324	33.33	[28.22; 38.76]
Otabil et al., 2023 (Tain District, Ghana) Baseline mf prev:40.7%	35	398	8.79	[6.20; 12.02]
Otabil et al., 2023 (Wenchi Municipality, Ghana) Baseline mf prev:48.1%	72	655	10.99	[8.70; 13.64]
**Random effects model**		.	**8.05**	**[5.55; 11.54]**
Heterogeneity: $I^2 = 98\%$, $\tau^2 = 1.3368$, $p = 0$				
**Ongoing transmission (without vector control)**				
Osei-Atweneboana et al., 2007 (Kintampo District, Ghana) Baseline nodule prev:NA%	4	235	1.70	[0.47; 4.30]
Katabarwa et al., 2008 (Bangangte, Cameroon) Baseline mf prev:78.3%	44	719	6.12	[4.48; 8.13]
Katabarwa et al., 2008 (Foumbot, Cameroon) Baseline mf prev:86%	162	719	22.53	[19.53; 25.76]
Katabarwa et al., 2008 (Bafang, Cameroon) Baseline mf prev:68%	14	719	1.95	[1.07; 3.25]
Katabarwa et al., 2008 (Kekem, Cameroon) Baseline mf prev:69.2%	19	719	2.64	[1.60; 4.10]
Katabarwa et al., 2008 (Bandja, Cameroon) Baseline mf prev:72.1%	70	719	9.74	[7.67; 12.14]
Katabarwa et al., 2008 (Kasese, Uganda) Baseline mf prev:70.7%	13	838	1.55	[0.83; 2.64]
Katabarwa et al., 2008 (Kisoro, Uganda) Baseline mf prev:53.2%	41	838	4.89	[3.53; 6.58]
Katabarwa et al., 2008 (Mbale, Uganda) Baseline mf prev:62.2%	13	838	1.55	[0.83; 2.64]
Katabarwa et al., 2008 (Moyo, Uganda) Baseline mf prev:80%	58	838	6.92	[5.30; 8.86]
Katabarwa et al., 2008 (Nebbi, Uganda) Baseline mf prev:98.9%	161	838	19.21	[16.60; 22.05]
Seidenfaden et al., 2001 (Vina du Nord River, Cameroon) Baseline mf prev:80%	16	70	22.86	[13.67; 34.45]
Abong et al., 2020 (Bafia Health District, Cameroon) Baseline nodule prev:NA%	105	388	27.06	[22.70; 31.78]
Abong et al., 2020 (Melong, Cameroon) Baseline mf prev:40%	70	599	11.69	[9.22; 14.53]
Katabarwa et al., 2013 (Nyagak-Bondo, Uganda) Baseline mf prev:97%	141	607	23.23	[19.92; 26.80]
Borsboom et al., 2003 (River Faleme, Senegal, Mali) Baseline mf prev:64%	17	341	4.99	[2.93; 7.86]
Borsboom et al., 2003 (Bafing, Guinea, Mali) Baseline mf prev:32%	31	341	9.09	[6.26; 12.66]
Borsboom et al., 2003 (River Bakoye, Guinea, Mali) Baseline mf prev:63%	31	341	9.09	[6.26; 12.66]
Djune-Yemeli et al., 2022 (Bafia, Cameroon) Baseline mf prev:24.4%	80	342	23.39	[19.01; 28.24]
Kamga et al., 2016 (Bafia, Cameroon) Baseline mf prev:90.3%	196	514	38.13	[33.91; 42.49]
Kamga et al., 2016 (Yabassi, Cameroon) Baseline mf prev:87.2%	109	242	45.04	[38.66; 51.54]
Kamga et al., 2017 (Bafang, Cameroon) Baseline mf prev:70.5%	10	328	3.05	[1.47; 5.54]
Kamga et al., 2017 (Bandja, Cameroon) Baseline mf prev:75.3%	3	136	2.21	[0.46; 6.31]
Kamga et al., 2017 (Bandjoun, Cameroon) Baseline mf prev:84.5%	17	371	4.58	[2.69; 7.24]
Kamga et al., 2017 (Bangangte, Cameroon) Baseline mf prev:81.3%	6	280	2.14	[0.79; 4.61]
Kamga et al., 2017 (Foumbot, Cameroon) Baseline mf prev:79%	18	410	4.39	[2.62; 6.85]
Kamga et al., 2017 (Kekem, Cameroon) Baseline mf prev:83.5%	6	231	2.60	[0.96; 5.57]
Kamga et al., 2017 (Massangam, Cameroon) Baseline mf prev:91.1%	65	302	21.52	[17.02; 26.59]
Osue Hudu., 2017 (Gurara river basin, Nigeria) Baseline nodule prev:NA%	16	438	3.65	[2.10; 5.86]
Otabil et al., 2019 (Nkoranza North district, Ghana) Baseline nodule prev:NA%	7	114	6.14	[2.50; 12.24]
Siewe et al., 2019 (Imo River Basin, Nigeria) Baseline nodule prev:NA%	4	1987	0.20	[0.05; 0.51]
Katabarwa et al., 2013 (Bafang, Cameroon) Baseline mf prev:63.9%	34	714	4.76	[3.32; 6.59]
Katabarwa et al., 2013 (Baham, Cameroon) Baseline mf prev:53.1%	21	189	11.11	[7.01; 16.48]
Katabarwa et al., 2013 (Bandja, Cameroon) Baseline mf prev:72.1%	18	84	21.43	[13.22; 31.74]
Katabarwa et al., 2013 (Bangangte, Cameroon) Baseline mf prev:76.7%	54	319	16.93	[12.98; 21.50]
Katabarwa et al., 2013 (Foumbot, Cameroon) Baseline mf prev:87.8%	89	317	28.08	[23.20; 33.37]
Katabarwa et al., 2013 (Kekem, Cameroon) Baseline mf prev:69.2%	8	163	4.91	[2.14; 9.44]
Katabarwa et al., 2013 (Penka-Michel, Cameroon) Baseline mf prev:56.1%	7	195	3.59	[1.46; 7.26]
Katabarwa et al., 2013 (Kouoptamo, Cameroon) Baseline nodule prev:NA%	5	168	2.98	[0.97; 6.81]
Katabarwa et al., 2013 (Malantouen, Cameroon) Baseline nodule prev:NA%	13	170	7.65	[4.13; 12.72]
Katabarwa et al., 2013 (Massangam, Cameroon) Baseline nodule prev:NA%	59	99	59.60	[49.26; 69.34]
Aza'ah et al., 2020 (Ndikinimeki, Cameroon) Baseline mf prev:54.7%	42	603	6.97	[5.07; 9.30]
Shintouo et al., 2020 (Bafang, Cameroon) Baseline nodule prev:NA%	9	203	4.43	[2.05; 8.25]
Shintouo et al., 2020 (Kekem, Cameroon) Baseline nodule prev:NA%	6	231	2.60	[0.96; 5.57]
Shintouo et al., 2020 (Massangam, Cameroon) Baseline nodule prev:NA%	53	123	43.09	[34.20; 52.32]
Nyagang et al., 2020 (Tombel, Cameroon) Baseline mf prev:5%	6	400	1.50	[0.55; 3.24]
Surakat et al., 2018 (Ogun, Nigeria) Baseline nodule prev:22.7%	83	481	17.26	[13.98; 20.94]
Katabarwa et al., 2011 (Rey-Bouba, Cameroon) Baseline mf prev:57.7%	3	119	2.52	[0.52; 7.19]
Katabarwa et al., 2011 (Touboro health district, Cameroon) Baseline nodule prev:NA%	32	406	7.88	[5.45; 10.94]
Sam-Wobo et al., 2012 (Ogun River System, Nigeria) Baseline nodule prev:NA%	296	1068	27.72	[25.05; 30.51]
Bakajika et al., 2018 (Massangam, Cameroon) Baseline mf prev:59.6%	78	680	11.47	[9.17; 14.11]
Forrer et al., 2021 (Meme River Basin, Cameroon) Baseline nodule prev:NA%	4198	9456	44.40	[43.39; 45.40]
Okon, O. E. and Ilugbiyin, E. F.., 2008 (Aninegeye, Nigeria) Baseline mf prev:11.9%	3	200	1.50	[0.31; 4.32]
Tekle et al., 2016 (Cross River, Nigeria) Baseline nodule prev:56.1%	356	7415	4.80	[4.33; 5.31]
Tekle et al., 2016 (Ebonyi, Nigeria) Baseline nodule prev:60.2%	45	3441	1.31	[0.96; 1.75]
Tekle et al., 2016 (Enugu Anambra, Nigeria) Baseline nodule prev:63%	135	4991	2.70	[2.27; 3.19]
Tekle et al., 2016 (Adjumani Mojo, Uganda) Baseline nodule prev:45.5%	98	5645	1.74	[1.41; 2.11]
Tekle et al., 2016 (Arua Nebbie, Uganda) Baseline nodule prev:87.3%	90	3894	2.31	[1.86; 2.83]
Tekle et al., 2016 (Adamawa II, Cameroon) Baseline nodule prev:57.5%	53	2816	1.88	[1.41; 2.45]
Tekle et al., 2016 (Centre 1, Cameroon) Baseline nodule prev:71.9%	1377	2576	53.45	[51.51; 55.40]
Tekle et al., 2016 (Littoral 2, Cameroon) Baseline nodule prev:59.9%	759	1576	48.16	[45.67; 50.66]
Tekle et al., 2016 (North Tchollire, Cameroon) Baseline nodule prev:54%	84	1377	6.10	[4.89; 7.50]
Tekle et al., 2016 (North Toubouro, Cameroon) Baseline nodule prev:81.9%	48	1230	3.90	[2.89; 5.14]
Tekle et al., 2016 (South West 1, Cameroon) Baseline nodule prev:71.7%	257	2159	11.90	[10.57; 13.34]
Tekle et al., 2016 (South West 2, Cameroon) Baseline nodule prev:65.6%	283	2055	13.77	[12.31; 15.34]
Tekle et al., 2016 (Western Province, Cameroon) Baseline nodule prev:86.4%	284	3239	8.77	[7.82; 9.80]
Tekle et al., 2016 (Basse-Kotto, CAR) Baseline nodule prev:59.7%	41	2758	1.49	[1.07; 2.01]
Tekle et al., 2016 (Uele, DRC) Baseline nodule prev:96.4%	595	1752	33.96	[31.74; 36.23]
Tekle et al., 2016 (Kafa, Shekka, Bench Maji, Ethiopia) Baseline nodule prev:47.4%	111	2167	5.12	[4.23; 6.14]
Tekle et al., 2016 (Lofa, Bong, Nimba, Liberia) Baseline nodule prev:27.6%	244	3554	6.87	[6.06; 7.75]
Tekle et al., 2016 (Ekiti, Nigeria) Baseline nodule prev:22.8%	36	1939	1.86	[1.30; 2.56]
Tekle et al., 2016 (Kwara, Nigeria) Baseline nodule prev:32.2%	29	2013	1.44	[0.97; 2.06]
Tekle et al., 2016 (Taraba, Nigeria) Baseline nodule prev:61.8%	214	1695	12.63	[11.08; 14.30]
Tekle et al., 2016 (Kilosa, Tanzania) Baseline nodule prev:25.1%	37	1488	2.49	[1.76; 3.41]
Tekle et al., 2016 (Mahenge, Tanzania) Baseline nodule prev:78.7%	176	1792	9.82	[8.48; 11.29]
Tekle et al., 2016 (Ruvuma, Tanzania) Baseline nodule prev:92.3%	104	2638	3.94	[3.23; 4.76]
Garms et al., 2015 (Toll bridge, Ghana) Baseline nodule prev:3.6%	19	50	38.00	[24.65; 52.83]
Garms et al., 2015 (Adwuman, Ghana) Baseline mf prev:47%	26	50	52.00	[37.42; 66.34]
Garms et al., 2015 (Amoafo, Ghana) Baseline mf prev:85%	15	32	46.88	[29.09; 65.26]
Garms et al., 2015 (Badowa, Ghana) Baseline mf prev:92%	6	30	20.00	[7.71; 38.57]
Wanji et al., 2015 (Manyu, Cameroon) Baseline mf prev:96.8%	251	761	32.98	[29.65; 36.45]
Wanji et al., 2015 (Mungo, Cameroon) Baseline mf prev:78.8%	408	995	41.01	[37.93; 44.13]
Wanji et al., 2015 (Meme, Cameroon) Baseline mf prev:78.8%	549	1041	52.74	[49.65; 55.81]
Hendy et al., 2018 (Mahenge, Tanzania) Baseline mf prev:60%	1034	12452	8.30	[7.82; 8.80]
Yaya et al., 2014 (Gami, Central African Republic) Baseline mf prev:88%	63	393	16.03	[12.54; 20.04]
Ekpo et al., 2022 (Amokwe, Nigeria) Baseline nodule prev:42%	23	45	51.11	[35.77; 66.30]
Ekpo et al., 2022 (Eziobodo, Nigeria) Baseline nodule prev:54%	15	45	33.33	[20.00; 48.95]
Ekpo et al., 2022 (Obinagu/Eziama, Nigeria) Baseline nodule prev:58%	16	50	32.00	[19.52; 46.70]
Ekpo et al., 2022 (Ugwuorie, Nigeria) Baseline nodule prev:42%	11	30	36.67	[19.93; 56.14]
Ekpo et al., 2022 (Umuezemanna, Nigeria) Baseline nodule prev:44%	7	15	46.67	[21.27; 73.41]
Ekpo et al., 2022 (Umunnakwe, Nigeria) Baseline nodule prev:44%	20	40	50.00	[33.80; 66.20]
Ekpo et al., 2022 (Abule Aje, Nigeria) Baseline nodule prev:4.8%	2	41	4.88	[0.60; 16.53]
Ekpo et al., 2022 (Abule Peter, Nigeria) Baseline nodule prev:4.8%	1	43	2.33	[0.06; 12.29]
Ekpo et al., 2022 (Adeaga, Nigeria) Baseline nodule prev:16%	9	32	28.12	[13.75; 46.75]
Ekpo et al., 2022 (Ibara Afon, Nigeria) Baseline nodule prev:30%	3	48	6.25	[1.31; 17.20]
Ekpo et al., 2022 (Ibaro, Nigeria) Baseline nodule prev:12%	12	42	28.57	[15.72; 44.58]
Ekpo et al., 2022 (Olokemeji, Nigeria) Baseline nodule prev:16%	6	28	21.43	[8.30; 40.95]
Efon-Ekanguouo et al., 2023 (Babeta, Cameroon) Baseline nodule prev:NA%	4	69	5.80	[1.60; 14.18]
Efon-Ekanguouo et al., 2023 (Biatsota, Cameroon) Baseline nodule prev:NA%	10	50	20.00	[10.03; 33.72]
Efon-Ekanguouo et al., 2023 (Ngongol, Cameroon) Baseline nodule prev:NA%	18	95	18.95	[11.63; 28.28]
Efon-Ekanguouo et al., 2023 (Nyamanga 1, Cameroon) Baseline nodule prev:NA%	10	65	15.38	[7.63; 26.48]
Efon-Ekanguouo et al., 2023 (Boneck, Cameroon) Baseline nodule prev:NA%	2	75	2.67	[0.32; 9.30]
Efon-Ekanguouo et al., 2023 (Kiboum 1, Cameroon) Baseline nodule prev:NA%	12	94	12.77	[6.77; 21.24]
Efon-Ekanguouo et al., 2023 (Kiboum 2, Cameroon) Baseline nodule prev:NA%	12	56	21.43	[11.59; 34.44]
**Random effects model**		.	**9.65**	**[7.56; 12.24]**
Heterogeneity: $I^2 = 99\%$, $\tau^2 = 1.8407$, $p = 0$				

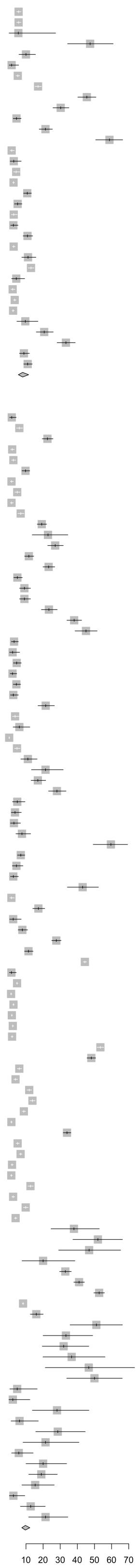

Figure S4: **Forest plots of microfilarial (mf) prevalence reported by studies conducting parasitological evaluations to measure the impact of interventions for records reporting ongoing onchocerciasis transmission, with or without vector control.** Records were classified as reporting ongoing transmission if mf prevalence ≥1% at the time of the evaluations. The references used are described in page 10. The grey boxes denote the effect sizes of studies and the horizontal lines are the 95%CIs.

Additional multivariable analysis

We conducted an additional (Poisson) meta-regression analysis on the association between microfilarial prevalence (number of skin-snip positive individuals out of total examined) at the time epidemiological evaluations were conducted after the intervention period being reported in the records providing parasitological results and the following variables: African region where the records originated from (West, Central, or East Africa) as per United Nations classification;[76] ecological features (savannah, forest, forest-savannah mosaic); larviciding of vector breeding sites (yes or no and duration); number of years of ivermectin treatment (<14 or ≥14 years); proportion of systematic non-adherence (≤5% as used in epidemiological models,[77,78] or >5%); years of continuous therapeutic coverage at ≥80% of eligible population (<10 or ≥10 years); baseline endemicity (i) hypoendemicity, ii) mesoendemicity, iii) hyperendemicity, and iv) holoendemicity as defined in the main text), and co-endemicity (not reported, with loiasis and/or with lymphatic filariasis). The results below present the log-risk ratios for the variables included in the final model, with the method of model selection as described in the main text.

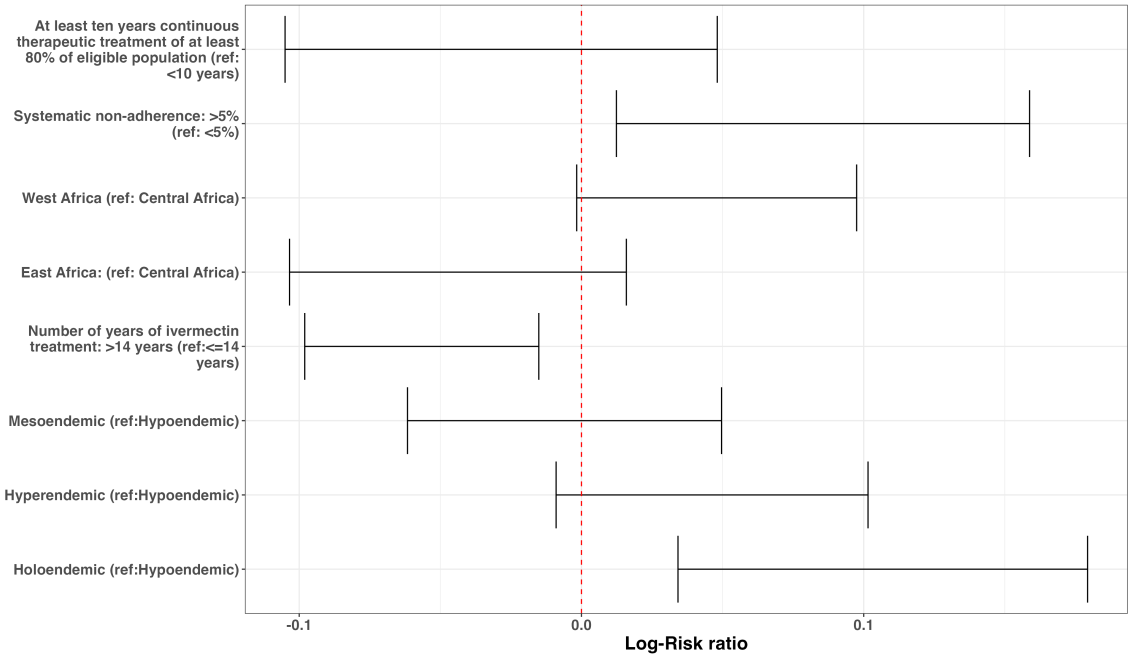

***Figure S5*:** Multivariate analysis of the factors associated with mf prevalence at the time epidemiological evaluations were conducted following the intervention period.

In foci that had received at least 14 years of ivermectin treatment, the risk of high mf prevalence was significantly lower (log-risk ratio: −0.06 [95%CI = −0.10– −0.02]) than in those with <14 years. Foci reporting systematic non-compliance >5% had a significantly higher risk of reporting high mf prevalence (log-risk ratio 0.09 [95%CI = 0.01–0.16]), compared to those with <5%. Taking hypoendemicity at baseline (pre-intervention) as the reference group, records that reported holoendemicity at baseline had significantly increased risk of presenting mf prevalence at the time of the epidemiological evaluation following the intervention period (log-risk ratio: 0.11 [95%CI = 0.03–0.18]).

Supplementary References

1 Abong RA, Amambo GN, Chounna Ndongmo PW, et al. Differential susceptibility of *Onchocerca volvulus* microfilaria to ivermectin in two areas of contrasting history of mass drug administration in Cameroon: relevance of microscopy and molecular techniques for the monitoring of skin microfilarial repopulation within six months of direct observed treatment. *BMC Infect Dis* 2020; **20:** 726.

2 Anosike JC, Dozie INS, Ameh GI, Ukaga CN, Nwoke BE, Nzechukwu CT, Udujih OS, Nwosu DC. The varied beneficial effects of ivermectin (Mectizan) treatment, as observed within onchocerciasis foci in south-eastern Nigeria. *Ann Trop Med Parasitol* 2007; **101:** 593–600.

3 Aza'ah RA, Sumo L, Ntonifor NH, Bopda J, Bamou RH, Nana-Djeunga HC. Point prevalence mapping reveals hotspot for onchocerciasis transmission in the Ndikinimeki Health District, Centre Region, Cameroon. *Parasit Vectors* 2020; **13:** 519.

4 Bakajika D, Senyonjo L, Enyong P, *et al.* On-going transmission of human onchocerciasis in the Massangam health district in the West Region of Cameroon: better understanding transmission dynamics to inform changes in programmatic interventions. *PLoS Negl Trop Dis* 2018; **12:** e0006904.

5 Biritwum NK, de Souza DK, Asiedu O, Marfo B, Amazigo UV, Gyapong JO. Onchocerciasis control in Ghana (1974–2016). *Parasit Vectors* 2021; **14:** 3.

6 Borsboom GJ, Boatin BA, Nagelkerke NJ, Agoua H, Akpoboua KL, Alley EW, Bissan Y, Renz A, Yameogo L, Remme JH, Habbema JD. Impact of ivermectin on onchocerciasis transmission: assessing the empirical evidence that repeated ivermectin mass treatments may lead to elimination/eradication in West-Africa. *Filaria J* 2003; **2:** 8.

7 Diawara L, Traoré MO, Badji A, Bissan Y, Doumbia K, et al. Feasibility of onchocerciasis elimination with ivermectin treatment in endemic foci in Africa: first evidence from studies in Mali and Senegal. *PLoS Negl Trop Dis* 2009; **3:** e479.

8 Djune-Yemeli L, Domché A, Nana-Djeunga HC, Donfo-Azafack C, Lenou-Nanga CG et al. Relationship between skin snip and Ov16 ELISA: two diagnostic tools for onchocerciasis in a focus in Cameroon after two decades of ivermectin-based preventive chemotherapy. *PLoS Negl Trop Dis* 2022; **16:** e0010380.

9 Dolo H, Coulibaly YI, Sow M, et al. Serological Evaluation of onchocerciasis and lymphatic filariasis elimination in the Bakoye and Falémé foci, Mali. *Clin Infect Dis* 2021; **72:** 1585–93.

10 Evans DS, Alphonsus K, Umaru J, et al. Status of onchocerciasis transmission after more than a decade of mass drug administration for onchocerciasis and lymphatic filariasis elimination in central Nigeria: challenges in coordinating the stop MDA decision. *PLoS Negl Trop Dis* 2014; **8:** e3113.

11 Forrer A, Wanji S, Obie ED, et al. Why onchocerciasis transmission persists after 15 annual ivermectin mass drug administrations in South-West Cameroon. *BMJ Glob Health* 2021; **6:** e003248.

12 Garms R, Badu K, Owusu-Dabo E, et al. Assessments of the transmission of *Onchocerca volvulus* by *Simulium sanctipauli* in the Upper Denkyira District, Ghana, and the intermittent disappearance of the vector. *Parasitol Res* 2015; **114:** 1129–37.

13 Gebrezgabiher G, Mekonnen Z, Yewhalaw D, Hailu A. Status of parasitological indicators and morbidity burden of onchocerciasis after years of successive implementation of mass distribution of ivermectin in selected communities of Yeki and Asosa districts, Ethiopia. *BMC Public Health* 2020; **20:** 1233.

14 Hendy A, Krüger A, Pfarr K, et al. The blackfly vectors and transmission of *Onchocerca volvulus* in Mahenge, south eastern Tanzania. *Acta Trop* 2018; **181:** 50–9.

15 Hernández-González A, Moya L, Perteguer MJ, et al. Evaluation of onchocerciasis seroprevalence in Bioko Island (Equatorial Guinea) after years of disease control programmes. *Parasit Vectors* 2016; **9:** 509.

16 Herrador Z, Garcia B, Ncogo P, et al. Interruption of onchocerciasis transmission in Bioko Island: Accelerating the movement from control to elimination in Equatorial Guinea. *PLoS Negl Trop Dis* 2018; **12:** e0006471.

17 Higazi TB, Zarroug IMA, Mohamed HA, et al. Interruption of *Onchocerca volvulus* transmission in the Abu Hamed focus, Sudan. *Am J Trop Med Hyg* 2013; **89:** 51–7.

18 Johanns SI, Gantin RG, Wangala B, et al. *Onchocerca volvulus*-specific antibody and cellular responses in onchocerciasis patients treated annually with ivermectin for 30 years and exposed to parasite transmission in central Togo. *PLoS Negl Trop Dis* 2022; **16:** e0010340.

19 Kamga GR, Dissak-Delon FN, Nana-Djeunga HC, et al. Still mesoendemic onchocerciasis in two Cameroonian community-directed treatment with ivermectin projects despite more than 15 years of mass treatment. *Parasit Vectors* 2016; **9:** 581.

20 Kamga GR, Dissak-Delon FN, Nana-Djeunga HC, et al. Important progress towards elimination of onchocerciasis in the West Region of Cameroon. *Parasit Vectors* 2017; **10:** 373.

21 Kamga GR, Dissak-Delon FN, Nana-Djeunga HC, et al. Audit of the community-directed treatment with ivermectin (CDTI) for onchocerciasis and factors associated with adherence in three regions of Cameroon. *Parasit Vectors* 2018; **11:** 356.

22 Katabarwa M, Eyamba A, Habomugisha P, et al. After a decade of annual dose mass ivermectin treatment in Cameroon and Uganda, onchocerciasis transmission continues. *Trop Med Int Health* 2008; **13:** 1196–203.

23 Katabarwa MN, Eyamba A, Nwane P, et al. Seventeen years of annual distribution of ivermectin has not interrupted onchocerciasis transmission in North Region, Cameroon. *Am J Trop Med Hyg* 2011; **85:** 1041–9.

24 Katabarwa MN, Walsh F, Habomugisha P, et al. Transmission of onchocerciasis in Wadelai focus of northwestern Uganda has been interrupted and the disease eliminated. *J Parasitol Res* 2012; **2012:** 748540.

25 Katabarwa MN, Eyamba A, Nwane P, et al. Fifteen years of annual mass treatment of onchocerciasis with ivermectin have not interrupted transmission in the West region of Cameroon. *J Parasitol Res* 2013a; **2013:** 420928.

26 Katabarwa MN, Lakwo T, Habomugisha P, et al. Transmission of *Onchocerca volvulus* continues in Nyagak-Bondo focus of northwestern Uganda after 18 years of a single dose of annual treatment with ivermectin. *Am J Trop Med Hyg* 2013b; **89:** 293–300.

27 Katabarwa M, Lakwo T, Habomugisha P, et al. Transmission of *Onchocerca volvulus* by *Simulium neavei* in Mount Elgon focus of eastern Uganda has been interrupted. *Am J Trop Med Hyg* 2014; **90:** 1159–66.

28 Katabarwa MN, Katamanywa J, Lakwo T, et al. The Imaramagambo onchocerciasis focus in southwestern Uganda: interruption of transmission after disappearance of the vector *Simulium neavei* and its associated freshwater crabs. *Am J Trop Med Hyg* 2016; **95:** 417–25.

29 Katabarwa MN, Zarroug IMA, Negussu N, et al. The Galabat-Metema cross-border onchocerciasis focus: the first coordinated interruption of onchocerciasis transmission in Africa. *PLoS Negl Trop Dis* 2020a; **14:** e0007830.

30 Katabarwa MN, Habomugisha P, Khainza A, et al. Elimination of *Simulium neavei*–transmitted onchocerciasis in Wambabya–Rwamarongo focus of western Uganda. *Am J Trop Med Hyg* 2020b; **103:** 1135–42.

31 Kogi E, Bulus W. The impact of ivermectin mass treatment on the prevalence of onchocerciasis by rapid epidemiological assessment in Galadimawa, Kaduna State, Nigeria. *Sci World J* 2008; **3:** 1–5.

32 Komlan K, Vossberg PS, Gantin RG, et al. *Onchocerca volvulus* infection and serological prevalence, ocular onchocerciasis and parasite transmission in northern and central Togo after decades of *Simulium damnosum* s.l. vector control and mass drug administration of ivermectin. *PLoS Negl Trop Dis* 2018; **12:** e0006312.

33 Koudou BG, Kouakou MM, Ouattara AF, et al. Update on the current status of onchocerciasis in Côte d'Ivoire following 40 years of intervention: progress and challenges. *PLoS Negl Trop Dis* 2018; **12:** e0006897.

34 Lakwo TL, Garms R, Rubaale T, et al. The disappearance of onchocerciasis from the Itwara focus, western Uganda after elimination of the vector *Simulium neavei* and 19 years of annual ivermectin treatments. *Acta Trop* 2013; **126:** 218–21.

35 Lakwo TL, Garms R, Tukahebwa E, et al. Successful interruption of the transmission of *Onchocerca volvulus* in Mpamba-Nkusi focus, Kibaale District, Uganda. *East Afr Med J* 2015; **92:** 401–7.

36 Lakwo T, Garms R, Wamani J, et al. Interruption of the transmission of *Onchocerca volvulus* in the Kashoya-Kitomi focus, western Uganda by long-term ivermectin treatment and elimination of the vector *Simulium neavei* by larviciding. *Acta Trop* 2017; **167:** 128–36.

37 Lakwo Luroni T, Gabriel M, Tukahebwa E, et al. The interruption of *Onchocerca volvulus* and *Wuchereria bancrofti* transmission by integrated chemotherapy in the Obongi focus, North Western Uganda. *PLoS One* 2017; **12:** e0189306.

38 Lamberton PHL, Cheke RA, Winskill P, et al. Onchocerciasis transmission in Ghana: persistence under different control strategies and the role of the simuliid vectors. *PLoS Negl Trop Dis* 2015; **9:** e0003688.

39 Moya L, Herrador Z, Ta-Tang TH, et al. Evidence for suppression of onchocerciasis transmission in Bioko Island, Equatorial Guinea. *PLoS Negl Trop Dis* 2016; **10:** e0004829.

40 Mweya CN, Kalinga AK, Kabula B, Malley KD, Ruhiso MH, Maegga BT. Onchocerciasis situation in the Tukuyu focus of southwest Tanzania after ten years of ivermectin mass treatment. *Tanzan Health Res Bull* 2007; **9:** 174–9.

41 Nyagang SM, Cumber SN, Cho JF, Keka EI, Nkfusai CN, Wepngong E, Tsoka-Gwegweni JM, Fokam EB. Prevalence of onchocerciasis, attitudes and practices and the treatment coverage after

15 years of mass drug administration with ivermectin in the Tombel health district, Cameroon. *Pan Afr Med J* 2020; **35:** 107.

42 Okon OE, Ilugbiyin EF. Evaluation of Mectizan efficacy in the treatment of onchocerciasis using microfilarial load in a community in south-eastern Nigeria. *Int J Biomed Hlth Sci* 2008; **4:** 1994–6.

43 Osei-Atweneboana MY, Eng JK, Boakye DA, Gyapong JO, Prichard RK. Prevalence and intensity of *Onchocerca volvulus* infection and efficacy of ivermectin in endemic communities in Ghana: a two-phase epidemiological study. *Lancet* 2007; **369:** 2021–9.

44 Osue HO, Inabo HI, Yakubu SE, et al. Impact of eighteen-year varied compliance to onchocerciasis treatment with ivermectin in sentinel savannah agrarian communities in Kaduna State of Nigeria. *ISRN Parasitol* 2013; **2013:** 960168.

45 Osue HO. Field-based evidence of single and few doses of annual ivermectin treatment efficacy in eliminating skin microfilaria load after a decade of intervention. *Ethiop J Health Sci* 2017; **27:** 129–38.

46 Otabil KB, Gyasi SF, Awuah E, et al. Prevalence of onchocerciasis and associated clinical manifestations in selected hypoendemic communities in Ghana following long-term administration of ivermectin. *BMC Infect Dis* 2019; **19:** 431.

47 Paulin HN, Nshala A, Kalinga A, Mwingira U, Wiegand R, Cama V, Cantey PT. Evaluation of onchocerciasis transmission in Tanzania: preliminary rapid field results in the Tukuyu Focus, 2015. *Am J Trop Med Hyg* 2017; **97:** 673–6.

48 Richards FO, Eigege A, Umaru J, et al. The interruption of transmission of human onchocerciasis by an annual mass drug administration program in Plateau and Nasarawa States, Nigeria. *Am J Trop Med Hyg* 2020; **102:** 582–92.

49 Sam-Wobo SO, Adeleke MA, Jayeola OA, et al. Epidemiological evaluation of onchocerciasis along Ogun river system, Southwest Nigeria. *J Vector Borne Dis* 2012; **49:** 101–4.

50 Seidenfaden R, Fischer A, Bonow I, Ekale D, Tanya V, Renz A. Combined benefits of annual mass treatment with ivermectin and cattle zooprophylaxis on the severity of human onchocerciasis in northern Cameroon. *Trop Med Int Health* 2001; **6:** 715–25.

51 Shintouo CM, Nguve JE, Asa FB, et al. Entomological assessment of *Onchocerca* species transmission by black flies in selected communities in the West Region of Cameroon. *Pathogens* 2020; **9:** 722.

52 Siewe Fodjo JN, Ukaga CN, Nwazor EO, et al. Low prevalence of epilepsy and onchocerciasis after more than 20 years of ivermectin treatment in the Imo River Basin in Nigeria. *Infect Dis Poverty* 2019; **8:** 8.

53 Sufi DA, Zainab T. Evaluation of onchocerciasis: A decade of post treatment with ivermectin in Zainabi and Ririwai Doguwa local government area of Kano State. *Adv Entomol* 2015; **3:** 1–5.

54 Surakat OA, Sam-Wobo SO, De Los Santos T, et al. Seroprevalence of onchocerciasis in Ogun State, Nigeria after ten years of mass drug administration with ivermectin. *Southern Afr J Infect Dis* 2018; **33:** 65–71.

55 Ta T-H, Moya L, Nguema J, et al. Geographical distribution and species identification of human filariasis and onchocerciasis in Bioko Island, Equatorial Guinea. *Acta Trop* 2018; **180:** 12–7.

56 Tekle AH, Elhassan E, Isiyaku S, et al. Impact of long-term treatment of onchocerciasis with ivermectin in Kaduna State, Nigeria: first evidence of the potential for elimination in the operational area of the African Programme for Onchocerciasis Control. *Parasit Vectors* 2012; **5:** 28.

57 Tekle AH, Zouré HGM, Noma M, Boussinesq M, Coffeng LE, Stolk WA, Remme JH. Progress towards onchocerciasis elimination in the participating countries of the African Programme for onchocerciasis control: epidemiological evaluation results. *Infect Dis Poverty* 2016; **5:** 66.

58 Traoré S, Wilson MD, Sima A, et al. The elimination of the onchocerciasis vector from the island of Bioko as a result of larviciding by the WHO African Programme for Onchocerciasis Control. *Acta Trop* 2009; **111:** 211–8.

59 Traore MO, Sarr MD, Badji A, et al. Proof-of-principle of onchocerciasis elimination with ivermectin treatment in endemic foci in Africa: final results of a study in Mali and Senegal. *PLoS Negl Trop Dis* 2012; **6:** e1825.

60 Wanji S, Kengne-Ouafo JA, Esum ME, et al. Situation analysis of parasitological and entomological indices of onchocerciasis transmission in three drainage basins of the rain forest of South West Cameroon after a decade of ivermectin treatment. *Parasit Vectors* 2015; **8:** 202.

61 Wilson NO, Badara Ly A, Cama VA, et al. Evaluation of lymphatic filariasis and onchocerciasis in three Senegalese districts treated for onchocerciasis with ivermectin. *PLoS Negl Trop Dis* 2016; **10:** e0005198.

62 Yaya G, Kobangué L, Kémata B, Gallé D, Grésenguet G. Élimination ou contrôle de l'onchocercose en Afrique ? Cas du village de Gami en République Centrafricaine. *Bull Soc Pathol Exot* 2014; **107:** 188–93.

63 Zarroug IM, Hashim K, ElMubark WA, et al. The first confirmed elimination of an onchocerciasis focus in Africa: Abu Hamed, Sudan. *Am J Trop Med Hyg* 2016; **95:** 1037–40.

64 Mshana MI, Silvestri V, Mushi V, Bonaventura WM, Tarimo D, et al. Burden and factors associated with onchocerciasis transmission among school-aged children after more than 20 years of Community Directed Treatment with Ivermectin in Ulanga district, Tanzania: a school-based cross-sectional study. *PLoS Glob Public Health* 2023; **3:** e0001919.

65 Atekem K, Dixon R, Wilhelm A, Biholong B, Oye J, et al. Evaluating the impact of alternative intervention strategies in accelerating onchocerciasis elimination in an area of persistent transmission in the West Region of Cameroon. *PLoS Negl Trop Dis* 2022; **16:** e0010591.

66 Otabil KB, Ankrah B, Bart-Plange EJ, et al. Prevalence of epilepsy in the onchocerciasis endemic middle belt of Ghana after 27 years of mass drug administration with ivermectin. *Infect Dis Poverty* 2023;12: 75.

67 Chikezie, F.M., Opara, K.N., Ubulom, P.M.E. et al. Onchocerciasis transmission status in some endemic communities of Cross River State, Nigeria after two decades of mass drug administration with ivermectin. *Sci Rep* 2023; **13**: 5413.

68 Ekpo, U.F., Eneanya, O.A., Nwankwo, E.N. et al. Persistence of onchocerciasis in villages in Enugu and Ogun states in Nigeria following many rounds of mass distribution of ivermectin. *BMC Infect Dis* 2022; **22**: 832.

69 Bhwana D, Amaral LJ, Mhina A, Hayuma PM, Francis F, et al. Impact of a bi-annual community-directed treatment with ivermectin programme on the incidence of epilepsy in an onchocerciasis-endemic area of Mahenge, Tanzania: a population-based prospective study. *PLoS Negl Trop Dis* 2023; **17**: e0011178.

70 Efon-Ekangouo A, Nana-Djeunga HC, Nzune-Toche N, Dongmo-Yemele R, Bopda J, et al. Impact of short-term discontinuation of ivermectin-based chemoprevention on onchocerciasis transmission in endemic settings with long history of mass drug administration. *PLoS Negl Trop Dis* 2023; **17:** e0011250.

71 Miri ES, Eigege A, Kahansim B, et al. Two Nigerian States (Plateau and Nasarawa) have eliminated transmission of human onchocerciasis: a report of post-ivermectin mass drug administration surveillance. *Am J Trop Med Hyg*. 2022;**108** :37–40.

72 Anagbogu, I.N., Saka, Y.A., Surakat, O. et al. Integrated transmission assessment surveys (iTAS) of lymphatic filariasis and onchocerciasis in Cross River, Taraba and Yobe States, Nigeria. *Parasit Vectors* 2022; **15**: 201.

73 Ekanya, R., Obie, E.D., Hamill, L. et al. The preparatory phase for ground larviciding implementation for onchocerciasis control in the Meme River Basin in South West Cameroon: the COUNTDOWN Consortium alternative strategy implementation trial. *Parasit Vectors* 2022; **15**: 219.

74 Domche A, Nwane PB, Nana Djeunga HC, et al. Status of *Onchocerca volvulus* (Spirurida: Onchocercidae) transmission and effect of climatic variables on the vector population dynamics after two decades of ivermectin-based preventive chemotherapy in the Mbam Valley (Centre Region, Cameroon). *J Med Entomol*. 2022;**59:** 2130–38.

75 Isiyaku S, Igbe M, Madaki S, et al. The interruption of transmission of onchocerciasis in Kaduna, Kebbi and Zamfara states, Nigeria: another milestone achievement. *Int Health*. 2022;**14 (Suppl 2):** ii43–ii54.

76 Stolk WA, Walker M, Coffeng LE, Basáñez MG, De Vlas SJ. Required duration of mass ivermectin treatment for onchocerciasis elimination in Africa: a comparative modelling analysis. *Parasit Vectors* 2015; **8:** 552.

77 United Nations Department of Economic and Social Affairs. World Population Prospects 2019 Volume I: Comprehensive Tables. 2019. https://population.un.org/wpp/Publications/Files/WPP2019_Volume-I_Comprehensive-Tables.pdf.

78 Cano J, Basáñez MG, O'Hanlon SJ, Tekle AH, Wanji S, Zouré HG, Rebollo MP, Pullan RL. Identifying co-endemic areas for major filarial infections in sub-Saharan Africa: seeking synergies and preventing severe adverse events during mass drug administration campaigns. *Parasit Vectors* 2018; **11:** 70.
